# High-Pressure and Thermal Pasteurization Applied to Smoothies Enhances (Poly)Phenol Bioaccessibility along the Gastrointestinal Tract

**DOI:** 10.1021/acs.jafc.4c09166

**Published:** 2025-06-11

**Authors:** Cristina Matías, Gema Pereira-Caro, María-José Sáiz-Abajo, Concepción Cid, Iziar A. Ludwig, María-Paz De Peña

**Affiliations:** † Centre for Nutrition Research and Department of Nutrition, Food Science and Physiology, Faculty of Pharmacy and Nutrition, University of Navarra, Pamplona 31008, Spain; ‡ National Centre for Food Technology and Safety (CNTA), San Adrián 31570, Spain; § Department of Agroindustry and Food Quality, Andalusian Institute of Agricultural and Fisheries Research and Training (IFAPA), Alameda del Obispo, Avda. Menéndez-Pidal, Córdoba 14004, Spain; ∥ Foods for Health Group, Instituto Maimónides de Investigación Biomédica de Córdoba (IMIBIC), Córdoba 14004, Spain; ⊥ IdiSNA, Navarra Institute for Health Research, Pamplona 31008, Spain

**Keywords:** (Poly)phenols, Fruit and vegetable based smoothies, High-Pressure Processing, Thermal Processing, Gastrointestinal bioaccessibility, Human gut metabolism

## Abstract

This research aimed to explore the effect of nonthermal (High-Pressure Processing) and thermal (High-Temperature Short-Time) pasteurization applied to a fruit and vegetable based smoothie on the bioaccessibility of (poly)­phenols and their biotransformation by human gut microbiota. Untreated and pasteurized smoothies were subjected to an *in vitro* gastrointestinal digestion and colonic fermentation. Native (poly)­phenols and their gut-related metabolites were analyzed by LC-MS. Both pasteurization techniques showed a protective effect against degradation of (poly)­phenols along the gastrointestinal tract. Thermal processing led to a more than 2-fold higher (poly)­phenol bioaccessibility (44%) compared to the untreated (17%) and nonthermally treated (21%) smoothies. Native (poly)­phenols were almost completely converted (83–87%) into low-molecular-weight catabolites by the gut microbiota in all smoothies. However, thermal treatment favored the generation of gut-related metabolites after colonic fermentation for 48 h compared to untreated and high pressure-treated smoothies, mainly due to the improved bioaccessibility observed after *in vitro* gastrointestinal digestion.

## Graphical abstract

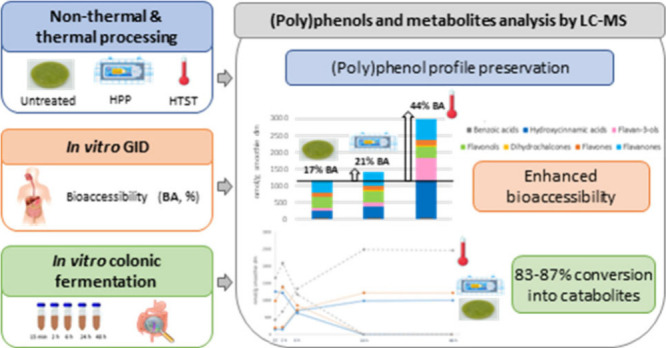

## 1 Introduction

Consumption of fruit and vegetable based beverages is increasing worldwide because of the growing consumer interest in the implications of diet on overall health.^1^ By 2030, the global functional beverage market is expected to grow to $1333.5 million, with a Compound Annual Growth Rate (CAGR) of 3.6% from 2024 to 2030.^2,3^ The benefits of fruits and vegetables are partly attributed to their richness in bioactive compounds, including (poly)­phenols with proven prevention against highly prevalent noncommunicable pathologies, such as neurodegenerative disorders, cardiovascular diseases, or cancer.^4,5^ Specifically, there is great interest in developing new products with enhanced bioaccessibility of these bioactive compounds.^6^ Moreover, these beverages are highly appreciated for their attractive organoleptic properties and convenience.

The World Health Organization suggests that one eat at least 400 g, or five portions, of fruits and vegetables per day to achieve health effects. However, the estimated daily intake is lower than the recommended values. In this regard, smoothies could fulfill the growing demand from this health-conscious population and provide an appealing and practical strategy to promote fruit and vegetable consumption.

Nonetheless, the health benefits of (poly)­phenols depend not only on the naturally present amounts in the plant matrices but also on the quantity that is accessible for absorption.^7^ To reach the systemic circulation and target tissues/cells and thus exert their health implications, it is essential that (poly)­phenols are first bioaccessible.^8^ Bioaccessibility is defined as the amount of a compound that is released during gastrointestinal digestion from the food matrix and becomes available for absorption.^9^ Further, (poly)­phenols may be subject to changes under digestive conditions, including pH and enzyme activities. The molecular structure and chemical properties of (poly)­phenols can strongly determine their stability throughout the gastrointestinal tract.^10^ In general, complex molecules are degraded to lower molecular weight compounds, which are more easily absorbed. For instance, the digestive environment could promote glycosidic/ester bond cleavage and the release of the aglycone. Similarly, highly polymerized compounds as procyanidins are poorly absorbable, and their degradation results in (epi)­catechin units. Overall, large amounts of (poly)­phenols are poorly absorbed in the upper gastrointestinal tract. Unabsorbed (poly)­phenols in the food matrix can reach the colon, where the human gut microbiota play a key role in the bioconversion of complex dietary (poly)­phenols into smaller microbial derivatives, which may even have higher bioactivity than their precursors.^11,12^

Food processing has been shown to influence the release of (poly)­phenols from the food matrix after gastrointestinal digestion, including the action of gut microbiota, due to modifications caused in tissue structures that could enhance (poly)­phenol bioaccessibility.^9,13,14^

Beverages based on fruits and vegetables are commercialized after a preservation treatment to extend their shelf life. Most studies related to preservation treatment of beverages assessed the impact of these treatments on safety, sensory properties, and the content of bioactive compounds, such as (poly)­phenols.^15–17^ However, limited literature is available on how pasteurization processing applied to smoothies impacts (poly)­phenol bioaccessibility and their conversion by colonic bacteria using human feces as a source of microbiota. Knowledge on bioaccessibility and microbiota conversion of (poly)­phenols present in processed fruit and vegetable based beverages is essential to understand their potential health benefits.

Considering that smoothies are commercialized after a preservation treatment, this research aimed to assess the impact of nonthermal and thermal pasteurization applied to a fruit and vegetable based smoothie on the bioaccessibility of (poly)­phenols after *in vitro* gastrointestinal digestion by LC-MS/MS. Additionally, the objective was to further explore the effect of these technologies on the colonic biotransformation of native (poly)­phenols and provide a better understanding to develop beverages rich in (poly)­phenols with enhanced bioaccessibility, including gut microbiota action, and thus food products with improved health benefits.

## 2 Materials and Methods

### 2.1 Chemicals and Reagents

Human saliva α-amylase (97.8 units per mg of solid), porcine pepsin (672 units per mg of solid), porcine pancreatin (8 × USP), porcine bile salts, CaCl_2_, Na_2_SO_4_·10H_2_O, urea, FeSO_4_·7H_2_O, and CoCl_2_·6H_2_O were obtained from Sigma-Aldrich (Darmstadt, Germany). KCl, KH_2_PO_4_, NaHCO_3_, NaCl, MgCl_2_·6H_2_O, (NH_4_)_2_CO_3_, HCl, CaCl_2_(H_2_O)_2_, NaOH, Na_2_HPO_4_·2H_2_O, MnSO_4_·H_2_O, ZnSO_4_·7H_2_O, CuSO·5H_2_O, and Mo­(NH_4_)_6_O_24_·4H_2_O were bought from PanReac Química S.L.U. (Barcelona, Spain).

(Poly)­phenols were named following the nomenclature proposed by Kay et al.^18^ Pure standards of 2,5-dihydroxybenzoic acid, 3,4-dihydroxybenzoic acid, 3,4,5-trihydroxybenzoic acid, 3,5-dimethoxy-4-hydroxybenzoic acid, 4′-hydroxycinnamic acid, 3′,4′-dihydroxycinnamic acid, 4′-hydroxy-3′-methoxycinnamic acid, 5-*O*-caffeoylquinic acid, 4-*O*-caffeoylquinic acid, 3,5-*O*-dicaffeoylquinic acid, 3,4-*O*-dicaffeoylquinic acid, 4,5-*O*-dicaffeoylquinic acid, 2-(3′,4′-dihydroxyphenyl)­ethanol, (−)-epicatechin, (+)-catechin, kaempferol, kaempferol-3-*O*-rutinoside, quercetin, quercetin-3-*O*-rhamnoside, quercetin-3-*O*-glucoside, quercetin-3-*O*-rutinoside, isorhamnetin, isorhamnetin-3-*O*-rutinoside, apigenin, apigenin-7-*O*-glucoside, apigenin-7-*O*-glucuronide, apigenin-7-*O*-rutinoside, apigenin-8-*C*-glucoside, apigenin-6,8-*C*-diglucoside, luteolin, luteolin-7-*O*-glucoside, luteolin-8-*C*-glucoside, luteolin-7-*O*-glucuronide, diosmetin, diosmetin-7-*O*-glucoside, naringenin-7-*O*-rutinoside, isosakuranetin-7-*O*-rutinoside, 4-methylbenzene-1,2-diol, 4-hydroxybenzoic acid, 3-(3′-hydroxyphenyl)­propanoic acid, phenylacetic acid, and naringenin were acquired from Sigma-Aldrich (Darmstadt, Germany).

Procyanidin dimer B_2_, procyanidin dimer B_1_, quercetin-3-*O*-arabinoside, kaempferol-7-*O*-glucoside, isorhamnetin-3-*O*-glucoside, phloretin-2′-*O*-glucoside, eriodictyol, and hesperetin-7-*O*-rutinoside were bought from Extrasynthese (Genay, France).

2-*O*-Caffeoyl-3-(3,4-dihydroxyphenyl)­lactic acid, 2,3-*O*-dicaffeoyltartaric acid, epigallocatechin, procyanidin trimer C_1_, kaempferol-3-*O*-glucuronide, quercetin-3-*O*-xyloside, quercetin-3-*O*-galactoside, phloretin, apigenin-7-(2-*O*-apiosylglucoside), diosmetin-7-*O*-rutinoside, eriodictyol-7-*O*-rutinoside, and hesperetin were supplied by MedChemExpress (Sollentuna, Sweden).

3-(3′,4′-Dihydroxyphenyl)­propanoic acid was acquired from Alfa Aesar (Kandel, Germany). 5-(3′,4′-Dihydroxyphenyl)-γ-valerolactone was obtained from Labclinics, SA (Barcelona, Spain). 5-(3′-Hydroxyphenyl)-γ-valerolactone and 5-(3′,4′-dihydroxyphenyl)­valeric acid were purchased from Toronto Research Chemicals (Toronto, Canada).

Acetonitrile and formic acid (both LC-MS grade) were purchased from Scharlau (Barcelona, Spain). Methanol (LC-MS grade) was supplied by PanReac AppliChem (Darmstadt, Germany).

### 2.2 Sample Preparation and Pasteurization Processing

Apples (*Malus domestica* cv. Granny Smith), green celery (*Apium graveolens*), green chicory (*Cichorium intybus* L.), and lemon (*Citrus limon*) were bought in a local market in Calahorra (Spain). Dried peppermint leaves (*Mentha piperita*) were acquired from the company Origeens (Estrées-Saint Denise, France). Fresh ingredients were washed before smoothie preparation. Green chicory was lyophilized in a freeze-dryer Cryodos-80 (Telstar, Terrasa, Spain). The smoothie was obtained by mixing Granny Smith apple juice (36.7 g/100 g), Granny Smith apple (30 g/100 g), green celery (30 g/100 g), lemon juice (2.7 g/100 g), dried green chicory (0.5 g/100 g), and dried peppermint leaves (0.1 g/100 g) at maximum speed for 1 min using a blender (Power Black Titanium 2000 Pro, Cecotec, Spain).

Two different pasteurization technologies were applied to the smoothie: High-Pressure Processing (HPP) and High-Temperature Short-Time Processing (HTST). A portion of the smoothie was left untreated to be used as a control. For HPP, the smoothie was immediately packaged in polyethylene bags and processed at 600 MPa for 6 min using a 10 L HPP unit (Idus HPP Systems S.L.U., Noain, Spain). For HTST treatment, the smoothie was subjected to 82 °C for 2.6 s in a continuous system comprised of tubular counterflow heat exchangers (Techni-Process, Thermalab III 20, Châteauneuf-le-Rouge, Francia) providing a flow rate of 31 L/h (*F*_70_ ≈ 6.6–9.5 min). The parameters were based on recommended thermal processing to ensure the absence of *Listeria monocytogenes* (at least a *F*_70_ ≈ 2 min). After pasteurization processes, smoothies were immediately cooled and lyophilized in a freeze-dryer (Cryodos-80, Telstar, Terrasa, Spain).

### 2.3 *In Vitro* Gastrointestinal Digestion

The static *in vitro* gastrointestinal digestion (GID) was performed following the method described by Brodkorb et al.^19^ The simulated digestion included three successive phases (oral, gastric, and intestinal) and was carried out under agitation in darkness at 37 °C. pH adjustments were made by adding 1 M HCl or 2 M NaOH based on previous pH-test experiments. The preparation of simulated salivary, gastric, and intestinal fluids (SSF, SGF, and SIF) is detailed in Table S1. For the oral phase, salivary α-amylase and SSF were used and incubated at pH 7 for 2 min. Next, for the gastric digestion, pepsin and SGF were added and kept at pH 3 for 2 h. Finally, the intestinal step was simulated by the addition of pancreatic enzymes, bile salts, and SIF at pH 7 for 2 h. Digested samples were immediately placed on ice to stop enzymatic activities, lyophilized in a freeze-dryer (Cryodos-80, Telstar, Terrasa, Spain), and stored at −18 °C until further analysis. Simulated gastrointestinal digestion was performed in duplicate for each smoothie. The extraction procedure, and subsequent analysis of (poly)­phenolic compounds, was performed in triplicate on each duplicate of the gastrointestinal digestion. Then, the duplicates were pooled as the colonic fermentation substrate.

### 2.4 *In Vitro* Colonic Fermentation

Digested smoothies were subjected to an *in vitro* colonic fermentation according to Del Burgo-Gutiérrez et al.,^13^ with slight modifications. A carbonate-phosphate buffer was employed as fermentation medium and prepared as previously reported by Mosele et al.^20^ Fresh feces were collected from four healthy volunteers (aged 24–58; BMI 18.5–24.9) who did not suffer from any intestinal diseases and had not been treated with antibiotics for at least the previous four months. Volunteers were on a (poly)­phenol-free diet for the 48 h prior to the collection of the fecal sample. The Research Ethics Committee of University of Navarra approved the protocol (no. 2023.077) and complied with the Declaration of Helsinki. After fecal deposition, samples were immediately kept under anaerobic conditions and used within 2 h. Feces were combined in the same proportion and then homogenized with the growth medium to obtain a 5% (w/v) fecal slurry using a stomacher for 1 min. Subsequently, 75 mL of fecal slurry was mixed with 937.5 mg of freeze-dried digested smoothies in glass bottles. Then, they were sealed using a rubber cap and aluminum seal, and an anaerobic environment was created using pure nitrogen. Bottles were placed on an orbital shaker at 60 rpm and incubated at 37 °C for 48 h. Aliquots of fermented samples (10 mL) were collected at 15 min, 2 h, 6 h, 24 and 48 h of incubation time, and fermentation was stopped by adding 60 μL of HCl. First sample aliquot was taken at minute 15, which corresponds to the time needed for mixing the digested sample with the fecal slurry, sealing the fermentation bottles, and flushing with nitrogen to generate the anaerobic atmosphere. Two types of control samples were incubated in parallel, one containing fecal slurry without digested smoothies and the other including growth medium and digested smoothies without fecal sample. Aliquots were cooled, lyophilized in a freeze-dryer (Cryodos-80, Telstar, Terrasa, Spain), and stored at −18 °C until further analysis. Simulated colonic fermentation was carried out in triplicate for each smoothie.

### 2.5 Extraction of (Poly)­Phenolic Compounds

The extraction of native (poly)­phenols and their gut-related metabolites from samples was conducted in accordance with Domínguez-Fernández et al.,^14^ with some modifications. In brief, 25 mg of freeze-dried samples was mixed with 0.5 mL of methanol/acidified Milli-Q water (0.1% formic acid) (80:20 v/v), vortexed, sonicated for 15 min, and then, centrifuged at 18626*g* for 10 min (Mikro 200, Hettich, Tuttlingen, Germany). After the first extraction, the residue was re-extracted with 0.25 mL of methanol/acidified Milli-Q water (0.1% formic acid) (50:50 v/v) following the same process. Both supernatants were combined and filtered through a 0.22 μm PVDF filter prior to LC-MS/MS analysis. Extractions were done in triplicate.

### 2.6 Analysis of (Poly)­Phenolic Compounds by LC-MS/MS

For bioaccessibility, samples were analyzed on an HPLC system model 1200 (Agilent Technologies, Palo Alto, CA, USA) coupled to a triple quadrupole linear ion trap mass spectrometer (3200 Q-TRAP, AB SCIEX, Madrid, Spain). (Poly)­phenols were separated using a CORTECS C18 column (3 mm × 75 mm, 2.7 μm) from Waters (Barcelona, Spain). Mobile phases consisted of acidified Milli-Q water (0.1% formic acid) (eluent A) and acetonitrile (eluent B). The column oven temperature was set at 30 °C, the injection volume was 5 μL, and the flow rate was 0.6 mL/min. The gradient elution was carried out as previously reported by Domínguez-Fernández et al.^21^

Mass spectrometric data were acquired using the ion multiple reaction monitoring (MRM) approach and the negative ionization mode. Targeted analysis was conducted for 57 compounds by comparing the retention time, the molecular ion, and the MS/MS fragmentation pattern with those of the pure standards (MS parameters are included in Table S2). Data were processed using Analyst version 1.6.3 (AB SCIEX). Results were expressed as mean ± standard deviation (SD) in nanomoles per gram of sample dry matter (nmol/g dm).

For *in vitro* colonic fermentation, samples were injected into a Vanquish UHPLC system connected to a Q-Exactive Orbitrap high-resolution mass spectrometer (HRMS) equipped with a heated ESI probe (Thermo Fisher Scientific, Waltham, MA, USA). Separation was achieved using a Hypersil GOLD C18 column (2.1 mm × 100 mm, 1.9 μm) from Thermo Fisher Scientific (Waltham, MA, USA). Mobile phases consisted of acidified Milli-Q water (0.1% formic acid) (eluent A) and acetonitrile (0.1% formic acid) (eluent B). The column oven temperature was maintained at 30 °C, the injection volume was set at 2 μL, and the flow rate was 0.25 mL/min. Elution was performed by a gradient in 22 min as follows: 0–1 min, 1% B; 1–5 min, 1–8% B; 5–15 min, 8–40% B; 15–17 min, 40–96% B; 17–19 min, 96% B; 19–20 min, 96–1% B, and then held constant until the end of the analysis.

Mass spectrometric data were conducted using the Full MS/data-dependent MS/MS acquisition (DDA) approach in negative ionization mode. Mass range was monitored at *m*/*z* 115–1500, resolution was 70,000 for full MS and 17,500 for data-dependent MS/MS experiments, and a NCE of 35 eV was applied in the HCD cell. The following HESI source parameters were set: sheath gas (nitrogen) flow rate, 40 arbitrary units; auxiliary gas (nitrogen) flow rate, 10 arbitrary units; sweep gas (nitrogen) flow rate, 1 arbitrary unit; spray voltage, −2.5 kV; capillary temperature, 320 °C; S-lens RF level, 55 V; and auxiliary gas heater temperature, 320 °C. Targeted compounds were analyzed by direct comparison with pure standards (MS parameters are detailed in Table S3). Data were processed using TraceFinder software version 5.1 (Thermo Fisher Scientific). Results were expressed as mean ± standard deviation (SD) in nmoles per gram of sample dry matter (nmol/g dm).

### 2.7 Bioaccessibility of (Poly)­Phenolic Compounds

Bioaccessibility (BA) was obtained as the percentage (%) of the concentration of each (poly)­phenol after *in vitro* gastrointestinal digestion compared to the initial concentration before digestion.

### 2.8 Statistical Analyses

Student’s *t* test was used to compare the concentration of (poly)­phenols before and after gastrointestinal digestion in untreated, HPP-treated, and HTST-treated smoothie. One-way analysis of variance (ANOVA) was employed to assess statistical significance (*p* ≤ 0.05) before and after gastrointestinal digestion among untreated, HPP-treated, and HTST-treated smoothies, followed by a post hoc Bonferroni test. Statistical analyses were performed using the STATA 17 software package (Stata Corp LLC, TX, USA).

## 3 Results and Discussion

Plant-based beverages undergo preservation processes to extend their shelf lives by reducing spoilage microorganisms and enzymatic activities. Food processing may modify (poly)­phenol contents and also modulate their bioaccessibility after gastrointestinal digestion, as well as their human gut microbiota metabolism.^13,14^ Thus, the impact of two common pasteurization technologies, nonthermal and thermal, on the bioaccessibility of (poly)­phenols from a fruit- and vegetable-based smoothie was evaluated. To this end, untreated and pasteurized (HPP and HTST) smoothies were subjected to a three-step *in vitro* gastrointestinal digestion, and (poly)­phenols were analyzed, before and after, by a LC-MS/MS targeted approach. Next, digested smoothies were submitted to an *in vitro* colonic fermentation and native (poly)­phenols and their gut-related metabolites were analyzed at different time points of colonic fermentation by a LC-HRMS.

### 3.1 Impact of HPP and HTST Processing on (Poly)­Phenols

From the 57 (poly)­phenols targeted in the LC-MS/MS method (Table S2), 43 (poly)­phenols were accurately identified and quantified in the smoothies (Table 2), encompassing the following subclasses: benzoic acids, hydroxycinnamic acids, flavan-3-ols, flavonols, dihydrochalcones, flavones, and flavanones (Table 1). After processing, HPP-treated and HTST-treated smoothies showed the same (poly)­phenolic profile as the unprocessed smoothie. Both pasteurization technologies (nonthermal and thermal) did not degrade the total amount of phenolic compounds (Table 1), and no significant differences (*p* > 0.05) among untreated and treated smoothies were found for all subclasses except for benzoic acids which showed statistically significantly lower values in the HTST-treated smoothie compared to the untreated smoothie. However, it should be stressed that just one representative of this subclass was detected (3,4-dihydroxybenzoic acid), and it was present in very low concentrations in all samples (3.35–6.26 nmol/g sample dm) accounting for not more than 0.1% of total (poly)­phenolic content. These findings indicate that both treatments are adequate for the preservation of the (poly)­phenol content of the freshly prepared fruit and vegetable smoothie. However, to exert their positive effect on health, polyphenols need to be accessible for absorption. Thus, the impact of processing on their bioaccessibility after *in vitro* gastrointestinal digestion was assessed.

**Table 1:** (Poly)­Phenols Grouped by Subclasses of Untreated, HPP-Treated, and HTST-Treated Smoothies before and after *In Vitro* Gastrointestinal Digestion and Their Bioaccessibility (BA, %)^a^

	untreated smoothie				HPP-treated smoothie				HTST-treated smoothie			
(Poly)phenolic compound	before digestion	after digestion	*p* ^a^	BA (%)	before digestion	after digestion	*p* ^a^	BA (%)	before digestion	after digestion	*p* ^a^	BA (%)
Benzoic acids	6.3 ± 0.6^A^	7.8 ± 1.0^a^	0.06	125	6.3 ± 0.4^A^	7.7 ± 0.7^a^	0.01	122	3.4 ± 0.2^B^	7.1 ± 0.5^a^	0.00	213
Hydroxycinnamic acids	1452.7 ± 9.1^A^	246.3 ± 5.1^a^	0.00	17	1388.2 ± 12.8^A^	358.6 ± 2.5^b^	0.00	26	1408.0 ± 11.4^A^	1145.8 ± 3.0^c^	0.00	81
**TOTAL NON-FLAVONOIDS**	1459.0 ± 8.7^A^	254.1 ± 4.7^a^	0.00	**17**	1394.5 ± 12.2^A^	366.3 ± 2.4^b^	0.00	**26**	1411.4 ± 10.9^A^	1152.9 ± 2.8^c^	0.00	**82**
Flavan-3-ols	3811.7 ± 23.3^A^	93.4 ± 1.3^a^	0.00	2	3657.2 ± 19.3^A^	162.0 ± 2.0^b^	0.00	4	3729.4 ± 35.8^A^	655.4 ± 2.2^c^	0.00	18
Flavonols	463.7 ± 3.1^A^	314.9 ± 3.3^a^	0.00	68	462.0 ± 3.8^A^	317.4 ± 1.9^b^	0.00	69	467.0 ± 1.7^A^	347.5 ± 0.9^c^	0.00	74
Dihydrochalcones	58.1 ± 3.2^A^	28.5 ± 0.7^a^	0.00	49	58.0 ± 1.3^A^	34.7 ± 1.2^b^	0.00	60	56.2 ± 1.4^A^	45.1 ± 1.2^c^	0.00	80
Flavones	187.1 ± 2.5^A^	125.4 ± 2.4^a^	0.00	67	189.2 ± 1.7^A^	133.8 ± 1.0^b^	0.00	71	188.5 ± 2.5^A^	149.4 ± 0.8^c^	0.00	79
Flavanones	809.4 ± 10.3^A^	339.6 ± 4.9^a^	0.00	42	818.4 ± 15.3^A^	386.3 ± 5.9^b^	0.00	47	810.7 ± 8.0^A^	608.6 ± 2.3^c^	0.00	75
**TOTAL FLAVONOIDS**	5330.0 ± 10.3^A^	901.8 ± 3.3^a^	0.00	**17**	5184.8 ± 10.3^A^	1034.2 ± 3.0^b^	0.00	**20**	5251.7 ± 14.4^A^	1806.1 ± 1.6^c^	0.00	**34**
**TOTAL PHENOLIC COMPOUNDS**	6789.0 ± 10.0^A^	1155.9 ± 3.6^a^	0.00	**17**	6579.3 ± 10.8^A^	1400.5 ± 2.9^b^	0.00	**21**	6663.1 ± 13.8^A^	2959.0 ± 1.9^c^	0.00	**44**

a Data are expressed as mean ± standard deviation (nmol/g sample dm).

b *p* ≤ 0.05 indicates significant differences before and after digestion in untreated, HPP-treated, and HTST-treated smoothie, respectively. Different upper-case letters for each row indicate significant differences (*p* ≤ 0.05) among untreated, HPP-treated, and HTST-treated smoothies before digestion. Different lower-case letters for each row indicate significant differences (*p* ≤ 0.05) among untreated, HPP-treated, and HTST-treated smoothies after digestion.

### 3.2 Bioaccessibility of (Poly)­Phenols after *In Vitro* Gastrointestinal Digestion

The action of digestive enzymes and pH changes throughout the gastrointestinal tract might induce changes in (poly)­phenols. The release of (poly)­phenols from the matrix and their absorption are highly influenced by chemical structure, such as molecular weight, degree of polymerization or occurrence as aglycone or conjugated derivative.^10,22,23^ Thus, the present research focused on the impact of the preservation processes on (poly)­phenol subclasses as well as on individual (poly)­phenolic compounds after *in vitro* digestion. (Poly)­phenolic compounds before and after *in vitro* gastrointestinal digestion and their bioaccessibility (BA, %) grouped by subclasses are reported in Table 1 and individual compounds are listed in Table 2. Chromatograms obtained from smoothie samples before and after *in vitro* gastrointestinal digestion are included in the Supporting Information (Figures S1–S3).

**Table 2:** (Poly)­Phenolic Profile of Untreated, HPP-Treated, and HTST-Treated Smoothies before and after *In Vitro* Gastrointestinal Digestion and Their Bioaccessibility (BA, %)^a^

	untreated smoothie			HPP-treated smoothie			HTST-treated smoothie		
(Poly)phenolic compound	before digestion	after digestion	BA (%)	before digestion	after digestion	BA (%)	before digestion	after digestion	BA (%)
NON-FLAVONOIDS									
Benzoic acids									
2,5-Dihydroxybenzoic acid	^a^	^a^		^a^	^a^		^a^	^a^	
3,4-Dihydroxybenzoic acid	6.3 ± 0.6	7.8 ± 1.0	125	6.3 ± 0.4	7.7 ± 0.7	122	3.4 ± 0.2	7.1 ± 0.5	213
3,4,5-Trihydroxybenzoic acid	^a^	^a^		^a^	^a^		^a^	^a^	
3,5-Dimethoxy-4-hydroxybenzoic acid	^a^	^a^		^a^	^a^		^a^	^a^	
Hydroxycinnamic acids									
4′-Hydroxycinnamic acid	2.2 ± 0.1	43.8 ± 0.2	1966	2.8 ± 0.1	45.3 ± 2.8	1629	2.3 ± 0.1	52.6 ± 1.7	2305
3′,4′-Dihydroxycinnamic acid	12.6 ± 0.3	7.4 ± 0.5	59	15.9 ± 1.3	13.1 ± 1.1	82	15.1 ± 0.5	64.1 ± 0.9	426
4′-Hydroxy-3′-methoxycinnamic acid	1.2 ± 0.1	21.1 ± 8.0	1722	1.2 ± 0.2	25.0 ± 1.5	2039	2.1 ± 0.2	27.5 ± 0.9	1313
2-*O*-Caffeoyl-3-(3′,4′-dihydroxyphenyl)lactic acid	123.6 ± 4.9	^b^		100.3 ± 2.5	3.2 ± 0.2	3	106.4 ± 5.5	48.2 ± 0.6	45
2,3-*O*-Dicaffeoyltartaric acid	208.6 ± 5.9	28.1 ± 0.9	13	198.5 ± 2.2	42.2 ± 0.7	21	202.7 ± 6.8	160.3 ± 4.5	79
5-*O*-Caffeoylquinic acid	1079.6 ± 27.8	131.7 ± 9.5	12	1045.4 ± 40.5	208.4 ± 5.7	20	1055.0 ± 35.1	720.1 ± 6.1	68
4-*O*-Caffeoylquinic acid	15.0 ± 0.8	14.2 ± 0.6	95	14.4 ± 0.4	21.3 ± 0.7	148	14.8 ± 0.3	72.9 ± 0.4	492
3,5-*O*-Dicaffeoylquinic acid	6.6 ± 0.8	^b^		6.4 ± 0.4	^b^		6.3 ± 0.2	^b^	
3,4-*O*-Dicaffeoylquinic acid	2.8 ± 0.1	^b^		2.8 ± 0.2	^b^		2.8 ± 0.3	^b^	
4,5-*O*-Dicaffeoylquinic acid	0.5 ± 0.1	^a^		0.5 ± 0.1	^a^		0.5 ± 0.0	^a^	
Phenylethanols									
2-(3′,4′-Dihydroxyphenyl)ethanol	^a^	^a^		^a^	^a^		^a^	^a^	
FLAVONOIDS									
Flavan-3-ols									
(−)-Epicatechin	403.6 ± 13.9	23.9 ± 0.3	6	383.7 ± 14.9	37.3 ± 1.7	10	402.2 ± 19.2	129.5 ± 2.6	32
(+)-Catechin	1412.0 ± 22.8	47.7 ± 1.2	3	1357.1 ± 6.5	83.1 ± 4.1	6	1410.3 ± 53.7	313.8 ± 2.9	22
Epigallocatechin	^a^	^a^		^a^	^a^		^a^	^a^	
Procyanidin B_2_ ((−)-epicat, (−)-epicat)	1278.2 ± 26.8	8.8 ± 2.2	1	1166.6 ± 34.0	21.5 ± 0.1	2	1231.2 ± 50.3	130.9 ± 0.4	11
Procyanidin B_1_ ((−)-epicat, (+)-cat)	443.3 ± 11.1	8.0 ± 1.6	2	414.8 ± 8.9	14.8 ± 0.8	4	446.9 ± 14.6	60.8 ± 1.0	14
Procyanidin C_1_ ((−)-epicat, (−)-epicat, (−)-epicat)	274.5 ± 34.0	5.0 ± 0.0	2	335.0 ± 19.1	5.3 ± 0.0	2	238.8 ± 20.1	20.3 ± 2.8	9
Flavonols									
Kaempferol	2.0 ± 0.0	5.2 ± 0.6	262	2.7 ± 0.2	5.4 ± 0.7	203	2.8 ± 0.0	8.1 ± 0.7	288
Kaempferol-7-*O*-glucoside	4.2 ± 0.4	3.0 ± 0.2	72	4.1 ± 0.3	3.3 ± 0.3	81	4.0 ± 0.3	2.9 ± 0.0	73
Kaempferol-3-*O*-glucuronide	195.3 ± 9.0	88.5 ± 7.3	45	182.7 ± 10.2	87.2 ± 4.9	48	191.0 ± 3.8	96.6 ± 1.7	51
Kaempferol-3-*O*-rutinoside	^a^	^a^		^a^	^a^		^a^	^a^	
Quercetin	2.3 ± 0.2	^a^		2.4 ± 0.1	^a^		2.5 ± 0.3	^a^	
Quercetin-3-*O*-arabinoside	4.4 ± 0.2	3.1 ± 0.1	69	4.8 ± 0.5	2.9 ± 0.0	60	4.7 ± 0.2	3.3 ± 0.2	71
Quercetin-3-*O*-xyloside	43.1 ± 2.4	32.4 ± 2.3	75	45.9 ± 3.2	33.3 ± 1.4	72	44.5 ± 1.8	36.4 ± 0.5	82
Quercetin-3-*O*-rhamnoside	104.1 ± 2.4	106.5 ± 6.3	102	108.9 ± 3.8	103.9 ± 2.8	95	106.7 ± 2.2	108.7 ± 2.1	102
Quercetin-3-*O*-glucoside	34.6 ± 1.5	22.9 ± 1.3	66	36.0 ± 2.7	23.6 ± 0.2	66	33.8 ± 1.0	27.2 ± 0.1	80
Quercetin-3-*O*-galactoside	51.2 ± 2.7	36.1 ± 3.3	70	52.9 ± 4.6	38.0 ± 0.4	72	53.8 ± 2.2	42.1 ± 0.9	78
Quercetin-3-*O*-rutinoside	8.9 ± 0.4	5.9 ± 0.8	66	8.5 ± 0.3	5.9 ± 0.2	69	8.8 ± 0.6	6.4 ± 0.1	73
Isorhamnetin	^a^	^a^		^a^	^a^		^a^	^a^	
Isorhamnetin-3-*O*-glucoside	^a^	^a^		^a^	^a^		^a^	^a^	
Isorhamnetin-3-*O*-rutinoside	13.6 ± 1.0	11.5 ± 0.8	85	13.1 ± 1.0	13.9 ± 0.1	106	14.4 ± 1.2	15.8 ± 0.4	110
Dihydrochalcones									
Phloretin	^a^	^a^		^a^	^a^		^a^	^a^	
Phloretin-2′-*O*-glucoside	58.1 ± 3.2	28.5 ± 0.7	49	58.0 ± 1.3	34.7 ± 1.2	60	56.2 ± 1.4	45.1 ± 1.2	80
Flavones									
Apigenin	3.2 ± 0.7	^a^		3.0 ± 0.2	^a^		2.3 ± 0.3	^a^	
Apigenin-7-*O*-glucoside	^a^	^a^		^a^	^a^		^a^	^a^	
Apigenin-7-*O*-glucuronide	1.5 ± 0.1	^a^		1.3 ± 0.1	^a^		1.3 ± 0.1	^a^	
Apigenin-7-*O*-rutinoside	17.6 ± 0.5	12.0 ± 0.3	68	17.5 ± 0.8	13.2 ± 0.6	75	17.2 ± 0.4	15.8 ± 0.3	92
Apigenin-7-(2-*O*-apiosylglucoside)	0.9 ± 0.1	^b^		0.8 ± 0.0	^b^		0.8 ± 0.0	^b^	
Apigenin-8-*C*-glucoside	^a^	^a^		^a^	^a^		^a^	^a^	
Apigenin-6,8-*C*-diglucoside	4.2 ± 0.2	^b^		3.8 ± 0.2	^b^		3.9 ± 0.1	^b^	
Luteolin	38.6 ± 1.1	68.2 ± 1.2	177	50.2 ± 2.4	64.9 ± 0.6	129	43.1 ± 4.8	62.8 ± 0.2	145
Luteolin-7-*O*-glucoside	2.9 ± 0.4	2.2 ± 0.3	75	3.2 ± 0.1	1.9 ± 0.4	61	2.8 ± 0.2	2.2 ± 0.2	81
Luteolin-8-*C*-glucoside	^a^	^a^		^a^	^a^		^a^	^a^	
Luteolin-7-*O*-glucuronide	69.0 ± 5.7	6.2 ± 0.6	9	61.3 ± 2.6	10.8 ± 0.6	18	65.2 ± 1.2	16.5 ± 0.6	25
Diosmetin	1.9 ± 0.3	^a^		2.1 ± 0.2	^a^		1.3 ± 0.1	^a^	
Diosmetin-7-*O*-glucoside	^a^	^a^		^a^	^a^		^a^	^a^	
Diosmetin-7-*O*-rutinoside	47.3 ± 5.3	36.8 ± 5.2	78	45.9 ± 3.8	42.9 ± 1.8	93	50.4 ± 6.3	52.0 ± 1.5	103
Flavanones									
Naringenin-7-*O*-rutinoside	6.2 ± 0.4	5.2 ± 0.1	83	6.1 ± 0.3	5.2 ± 0.2	84	5.8 ± 0.2	5.9 ± 0.0	101
Isosakuranetin-7-*O*-rutinoside	1.8 ± 0.2	1.5 ± 0.3	81	1.8 ± 0.1	1.4 ± 0.1	82	1.8 ± 0.1	1.7 ± 0.0	95
Eriodictyol	34.1 ± 1.9	17.8 ± 2.5	52	40.7 ± 1.6	16.3 ± 0.3	40	32.8 ± 3.1	18.0 ± 0.0	55
Eriodictyol-7-*O*-rutinoside	645.1 ± 24.8	238.1 ± 11.1	37	650.9 ± 37.5	287.3 ± 13.1	44	655.8 ± 19.1	500.5 ± 3.9	76
Hesperetin	4.3 ± 0.5	3.0 ± 0.3	71	4.8 ± 0.3	^b^		3.8 ± 0.1	^b^	
Hesperetin-7-*O*-rutinoside	117.8 ± 3.9	74.0 ± 3.7	63	114.1 ± 2.4	76.1 ± 1.2	67	110.7 ± 2.1	82.5 ± 3.2	74

a Data are expressed as mean ± standard deviation (nmol/g sample dm).

b Not detected.

c Below the limit of quantification.

Untreated smoothies showed a considerable decrease in (poly)­phenols after *in vitro* gastrointestinal digestion, leading to poor bioaccessibility of total phenolic compounds (17%). Although a decrease in concentrations was found in all subclasses in the digested smoothie (Table 1), except for the benzoic acids, GID modulated bioaccessibility differently depending on the subclass.

Hydroxycinnamic acids were the main non-flavonoids accounting for 17% of the total amount of (poly)­phenols in the smoothie. They were, overall, highly degraded after GID and showed low bioaccessibility of 17%. Among individual hydroxycinnamic acids (Table 2), some low molecular derivatives, such as 4′-hydroxycinnamic acid and 4′-hydroxy-3′-methoxycinnamic acid, were present in the smoothie in low quantities, but their concentration increased after GID. In contrast, higher molecular weight compounds, such as 2-*O*-caffeoyl-3-(3′,4′-dihydroxyphenyl)­lactic acid, 2,3-*O*-dicaffeoyltartaric acid, and 5-*O*-caffeoylquinic acid, were considerably decreased and showed very low or no bioaccessibility (0%, 13%, and 12%, respectively). These last three phenolics were the major hydroxycinnamic acids in the smoothie and strongly determined the bioaccessibility of non-flavonoids. These outcomes are in good agreement with previous studies on bioaccessibility of hydroxycinnamic rich foods which reported poor bioaccessibility of untreated/raw samples.^14,24^

Flavonoids present in the smoothie include flavan-3-ols, flavonols, dihydrochalcones, flavones, and flavanones (Table 1). Among them, flavan-3-ols were the most abundant, accounting for 57% of the total (poly)­phenols in the smoothie and were drastically decreased after GID and were by far the least bioaccessible flavonoids (2%). All individual flavan-3-ols showed very low bioaccessibility (Table 2), but monomeric (−)-epicatechin and (+)-catechin (6% and 3% BA) were slightly more bioaccessible than di- and trimeric procyanidins (1–2% BA) suggesting an impact of the molecular weight/polymerization degree. The extremely low bioaccessibility of flavan-3-ols found in the digested smoothie is in accordance with values reported in other matrices containing these compounds, such as guarana,^10^ cocoa-based beverages,^25^ or apple.^26,27^ The poor recovery or even complete degradation of flavan-3-ols after GID may be attributed to their instability in the pH environment of the gastrointestinal tract, particularly at neutral pH of the intestinal phase, as well as of epimerization, polymerization, and autoxidation reactions stated in these studies.

Flavanones accounted for 15% of (poly)­phenols in the smoothie and showed a bioaccessibility of 42% after GID, ranging, however, from 37 to 83% for individual derivatives (Table 2). It should further be stressed that the major flavanone, eriodyctiol-7-*O*-rutinoside, was the compound with the lowest bioaccessibility (37%). Their different stability throughout the GID environment may be explained by the kind of aglycone, along with their possible conversion into chalcones under alkaline conditions.^28,29^

Flavonols and flavones were minor subclasses present in the smoothie and showed similar total bioaccessibilities of 67–68% (Table 1). Among flavonols, the major quercetin representative (quercetin-3-*O*-rhamnoside) showed 100% bioaccessibility, while the major kaempferol derivative, kaempferol-3-*O*-glucuronide, was notably degraded to 45%. Contrary to other studies on their bioaccessibility^30^ no increase of their aglycones was observed. Discrepancies between the results could be because they used a dynamic model rather than a static one. Similar to flavonols, flavones apigenin-7-*O*-rutinoside and diosmetin-7-*O*-rutinoside decreased after GID (68% and 78% BA) without liberation of their aglycones. In contrast, luteolin-7-*O*-glucuronide was strongly degraded (9% BA), but with an increase of luteolin in line with previous studies on pepper rich in luteolin derivatives.^13^

Among dihydrochalcones, typical phenolics of apples, the only representative detected, phloretin-2′-*O*-glucoside, seemed to be unstable after digestion and presented a bioaccessibility of 49%. These findings are in line with other studies on their bioaccessibility in apples which found considerable degradation of phloretins after GID.^26,30^

Regarding pasteurization treatments, the application of both technologies exerted a positive effect on (poly)­phenol stability in the gastrointestinal environment (Table 1), resulting in an enhanced bioaccessibility of total phenolic compounds (HPP, 21%; and HTST, 44%) compared to the untreated smoothie (17%). This is consistent with previous works where food processing improved the (poly)­phenol bioaccessibility due to a protective effect against enzymatic and pH-dependent degradation.^14,31^

Comparing the two technologies, HTST processing had a marked influence on the three major phenolic acids 2-*O*-caffeoyl-3-(3′,4′-dihydroxyphenyl)­lactic acid, 2,3-*O*-dicaffeoyltartaric acid, and 5-*O*-caffeoylquinic acid, resulting in outstanding preservation of hydroxycinnamic acid, the second most abundant subclass in the smoothie. Their bioaccessibility increased from 17% in the untreated smoothie to 81% in the HTST-treated one and increased also notably compared to pressure pasteurization (26% BA).

In the case of flavan-3-ols, HTST increased bioaccessibility up to 18% compared to 4% in HPP. This is of great relevance as flavan-3-ols were the most abundant phenolics in the smoothie and, unfortunately, the most negatively affected by *in vitro* GID (untreated, 2% BA). As observed in untreated smoothie, bioaccessibility was determined by the degree of polymerization, and (epi)­catechins suffered lower degradation than procyanidins (Table 2).

Pasteurization processing seemed to positively modulate the bioaccessibility of flavanones (untreated, 42% BA). Both technologies increased their bioaccessibility, but this was more pronounced in HTST treatment (47% HPP vs 75% HTST) especially because of the improved bioaccessibility of eriodyctiol-7-*O*-rutinoside, the main flavanone in the smoothies. Similarly, He et al.^32^ found that high-pressure homogenization had little impact on phenolic bioaccessibility in orange juice, while thermal treatments enhanced it considerably.

Bioaccessibility of minor subclasses, flavonols and flavones, was only minimally enhanced by processing, showing a slight increase from 68 to 74% and 67 to 79%, respectively. In the case of dihydrochalcones, the improvement of bioaccessibility was more pronounced (untreated, 49%; HPP, 60%; and HTST, 80%); however, this subclass accounts only for 2% of the total (poly)­phenols in the smoothie.

In summary, bioaccessibility was positively modulated by both pasteurization processes (untreated, 17%; HPP, 21%; HTST, 44%). Our results are aligned with those previously reported,^9,30,32,33^ which stated that food processing, including HPP and thermal processing, may improve (poly)­phenol bioaccessibility due to cell wall disruption and softening favoring the release of these compounds from the food matrix. Contrary to the bad reputation of thermal treatments, pasteurization led further to a more than 2-fold higher (poly)­phenol bioaccessibility compared to untreated and HPP-treated smoothies. Smoothie preparation already requires blending of raw materials and juice squeezing, which could induce a similar effect on tissues to HPP which could explain the lower improvement of (poly)­phenol bioaccessibility in the HPP-treated smoothie compared to the HTST-treated one. The considerably higher protective effect on (poly)­phenols exerted by thermal treatment may further be explained by the incorporation of phenolic compounds into Maillard reaction products such as melanoidins, which are formed during heat treatment. The formed complexes may prevent (poly)­phenol degradation due to the adverse gastrointestinal conditions (pH, and enzyme activity) on (poly)­phenol stability as proposed by Juániz et al. and De Santiago et al.^31,34,35^

### 3.3 Effect of Human Gut Microbiota Metabolism on (Poly)­Phenols

(Poly)­phenols present after GID may be partly absorbed in the upper gastrointestinal tract. However, it is widely known that substantial amounts of (poly)­phenols reach the colon and may be metabolized by the gut microbiota into low-molecular-weight compounds. These microbial derivatives might exert health benefits in the colon or be more easily absorbed than native (poly)­phenols and enter systemic circulation.^36–39^

In the present research, digested smoothies were used as substrate of an *in vitro* colonic fermentation with fecal samples for 48 h. From the 75 (poly)­phenols targeted in the LC-HRMS method (Table S3), 32 (poly)­phenols were accurately identified and quantified in the fermented samples (Table 4). The total sums of native (poly)­phenols and microbial metabolites found after 15 min, 2 h, 6 h, 24 h, and 48 h of incubation grouped by subclasses are reported in Table 3, while individual compounds are listed in Table 4. Example chromatograms obtained from HTST-treated samples after 15 min and 2, 6, 24, and 48 h of incubation are included in Supporting Information (Figure S4).

**Table 3:** Native (Poly)­Phenols and Gut-Related Metabolites of Untreated, HPP-Treated, and HTST-Treated Smoothies Grouped by Subclasses^a^

(Poly)phenolic compound	untreated smoothie	HPP-treated smoothie	HTST-treated smoothie
NON-FLAVONOIDS			
Total Benzene diols^a^			
t 15 min	^b^	^b^	^b^
t 2 h	^b^	^b^	^b^
t 6 h	189.5 ± 21.9	142.6 ± 16.6	145.1 ± 12.2
t 24 h	394.8 ± 26.3	437.0 ± 19.9	1017.0 ± 137.6
t 48 h	753.0 ± 94.9	1163.8 ± 165.8	3711.2 ± 364.3
Total Benzoic acids			
t 15 min	^b^	2.7 ± 0.7	6.5 ± 0.3
t 2 h	0.6 ± 0.0	0.2 ± 0.0	18.0 ± 2.5
t 6 h	259.6 ± 2.7	285.0 ± 13.5	345.2 ± 3.9
t 24 h	195.4 ± 8.3	232.5 ± 12.3	297.5 ± 16.7
t 48 h	97.2 ± 4.1	145.6 ± 6.8	163.1 ± 4.2
Total Hydroxycinnamic acids			
t 15 min	147.8 ± 6.6	186.2 ± 6.8	416.1 ± 14.2
t 2 h	65.1 ± 3.3	157.2 ± 7.8	500.1 ± 20.2
t 6 h	^c^	^c^	31.7 ± 2.4
t 24 h	^c^	^c^	^c^
t 48 h	^c^	^c^	^c^
Total Phenylpropanoic acids			
t 15 min	^b^	^b^	^b^
t 2 h	83.4 ± 7.7	51.1 ± 3.8	147.9 ± 4.6
t 6 h	423.5 ± 13.8	430.0 ± 15.0	953.2 ± 30.4
t 24 h	682.1 ± 48.3	871.9 ± 60.8	1816.3 ± 73.5
t 48 h	770.9 ± 35.6	921.0 ± 51.1	1987.3 ± 45.7
Total Phenylacetic acids^a^			
t 15 min	105.9 ± 7.7	180.3 ± 6.0	440.8 ± 24.5
t 2 h	79.1 ± 11.2	150.0 ± 9.0	539.3 ± 49.6
t 6 h	^b^	^b^	^b^
t 24 h	^b^	^b^	^b^
t 48 h	^b^	^b^	^b^
Total Phenyl-γ-valerolactones			
t 15 min	^c^	^c^	^c^
t 2 h	^c^	^c^	^c^
t 6 h	^c^	^c^	^c^
t 24 h	16.9 ± 0.3	26.9 ± 0.3	276.8 ± 14.8
t 48 h	21.8 ± 0.3	38.2 ± 2.2	191.8 ± 5.2
Total 5-Phenylvaleric acids			
t 15 min	^b^	7.1 ± 0.0	7.4 ± 0.0
t 2 h	^b^	7.6 ± 0.1	7.3 ± 0.3
t 6 h	7.6 ± 0.3	7.7 ± 0.4	7.6 ± 0.2
t 24 h	9.9 ± 2.5	8.5 ± 0.2	10.9 ± 0.8
t 48 h	10.0 ± 2.7	8.8 ± 0.3	13.1 ± 0.8
Total Phenylethanols			
t 15 min	^b^	^b^	^b^
t 2 h	^b^	^b^	^b^
t 6 h	^c^	^c^	^c^
t 24 h	82.5 ± 0.5	80.1 ± 1.1	83.6 ± 1.5
t 48 h	98.9 ± 5.1	101.6 ± 11.8	108.0 ± 7.2
TOTAL NON-FLAVONOIDS			
t 15 min	147.8 ± 5.4	196.0 ± 4.8	430.0 ± 11.6
t 2 h	149.1 ± 5.3	216.0 ± 5.5	673.4 ± 14.5
t 6 h	690.7 ± 7.6	722.8 ± 11.9	1337.7 ± 14.6
t 24 h	986.9 ± 22.2	1219.8 ± 29.5	2485.1 ± 33.5
t 48 h	998.9 ± 16.2	1216.1 ± 24.7	2463.3 ± 19.9
FLAVONOIDS			
Total Flavan-3-ols			
t 15 min	37.9 ± 2.0	34.9 ± 1.9	229.3 ± 10.0
t 2 h	30.8 ± 2.0	40.4 ± 2.4	359.7 ± 7.0
t 6 h	6.9 ± 0.5	21.0 ± 0.5	70.4 ± 1.4
t 24 h	^c^	8.1 ± 0.6	9.0 ± 1.4
t 48 h	^c^	^c^	8.6 ± 04
Total Flavonols			
t 15 min	103.3 ± 2.5	84.8 ± 1.5	119.3 ± 5.9
t 2 h	92.1 ± 3.7	93.8 ± 3.1	114.2 ± 3.4
t 6 h	26.0 ± 2.5	40.8 ± 1.8	43.4 ± 0.7
t 24 h	^c^	^c^	^c^
t 48 h	^c^	^c^	^c^
Total Flavones			
t 15 min	78.6 ± 1.5	59.5 ± 2.9	87.6 ± 1.7
t 2 h	76.7 ± 4.5	79.5 ± 5.9	103.6 ± 6.0
t 6 h	41.1 ± 5.9	59.6 ± 0.6	76.0 ± 1.9
t 24 h	^c^	^c^	^c^
t 48 h	^c^	^c^	^c^
Total Flavanones			
t 15 min	1033.3 ± 10.4	805.0 ± 4.0	1215.5 ± 31.8
t 2 h	1013.7 ± 58.5	1168.5 ± 86.2	1499.7 ± 85.2
t 6 h	554.5 ± 65.9	726.2 ± 5.6	980.3 ± 50.2
t 24 h	^c^	^c^	^c^
t 48 h	^c^	^c^	^c^
TOTAL FLAVONOIDS			
t 15 min	1253.2 ± 6.4	983.9 ± 3.0	1651.7 ± 19.4
t 2 h	1213.3 ± 32.2	1382.3 ± 49.9	2077.2 ± 42.9
t 6 h	628.5 ± 38.1	847.6 ± 3.0	1170.1 ± 22.5
t 24 h	^c^	8.1 ± 0.6	9.0 ± 1.4
t 48 h	^c^	^c^	8.6 ± 0.4
TOTAL PHENOLIC COMPOUNDS			
t 15 min	1400.9 ± 6.5	1179.9 ± 3.5	2081.7 ± 17.6
t 2 h	1362.4 ± 26.4	1598.3 ± 38.8	2750.5 ± 34.5
t 6 h	1319.2 ± 26.5	1570.4 ± 8.4	2507.8 ± 19.2
t 24 h	986.9 ± 22.2	1227.9 ± 28.0	2494.1 ± 32.1
t 48 h	998.9 ± 16.2	1216.1 ± 24.7	2471.9 ± 19.1

a Data are expressed as mean ± standard deviation (nmol/g sample dm).

b Phenolic compounds derived from other sources and excluded for the total (poly)­phenols calculation.

c Not detected.

d Below the limit of quantification.

**Table 4:** Native (Poly)­Phenols and Gut-Related Metabolites of Untreated, HPP-Treated, and HTST-Treated Smoothies^a^

(Poly)phenolic compound	untreated smoothie	HPP-treated smoothie	HTST-treated smoothie
NON-FLAVONOIDS			
Benzene diols			
4-Methylbenzene-1,2-diol^a^			
t 15 min	^b^	^b^	^b^
t 2 h	^b^	^b^	^b^
t 6 h	189.5 ± 21.9	142.6 ± 16.6	145.1 ± 12.2
t 24 h	394.8 ± 26.3	437.0 ± 19.9	1017.0 ± 137.6
t 48 h	753.0 ± 94.9	1163.8 ± 165.8	3711.2 ± 364.3
Benzoic acids			
4-Hydroxybenzoic acid			
t 15 min	^b^	^b^	^b^
t 2 h	^b^	^b^	^b^
t 6 h	201.9 ± 3.1	230.5 ± 23.2	243.2 ± 6.7
t 24 h	124.5 ± 14.9	154.4 ± 20.3	181.0 ± 32.2
t 48 h	12.2 ± 0.7	47.8 ± 4.7	45.4 ± 6.8
2,5-Dihydroxybenzoic acid			
t 15 min	^b^	^b^	^b^
t 2 h	0.6 ± 0.0	0.2 ± 0.0	^b^
t 6 h	26.7 ± 3.5	23.6 ± 3.1	28.1 ± 2.5
t 24 h	26.5 ± 0.2	24.8 ± 1.6	23.7 ± 1.3
t 48 h	45.0 ± 2.3	43.3 ± 6.9	46.4 ± 3.1
3,4-Dihydroxybenzoic acid			
t 15 min	^b^	2.7 ± 0.7	6.5 ± 0.3
t 2 h	^b^	^b^	18.0 ± 2.5
t 6 h	^b^	^b^	41.3 ± 3.0
t 24 h	^b^	^b^	40.8 ± 6.0
t 48 h	^b^	^b^	9.1 ± 0.9
3,4,5-Trihydroxybenzoic acid			
t 15 min	^b^	^c^	^b^
t 2 h	^c^	^c^	^c^
t 6 h	31.0 ± 0.6	30.9 ± 0.3	32.6 ± 1.2
t 24 h	44.3 ± 7.5	53.3 ± 5.9	52.0 ± 6.5
t 48 h	40.1 ± 6.8	54.5 ± 8.3	62.1 ± 4.0
Hydroxycinnamic acids			
4′-Hydroxycinnamic acid			
t 15 min	90.3 ± 8.0	74.7 ± 9.3	84.1 ± 13.0
t 2 h	34.8 ± 3.5	72.0 ± 10.3	78.2 ± 4.5
t 6 h	^c^	^c^	^c^
t 24 h	^c^	^c^	^c^
t 48 h	^c^	^c^	^c^
3′,4′-Dihydroxycinnamic acid			
t 15 min	57.4 ± 4.8	111.5 ± 2.0	304.4 ± 25.3
t 2 h	30.3 ± 3.2	85.2 ± 4.0	382.4 ± 40.0
t 6 h	^b^	^b^	^b^
t 24 h	^b^	^b^	^b^
t 48 h	^b^	^b^	^b^
2-*O*-Caffeoyl-3-(3′,4′-dihydroxyphenyl)lactic acid			
t 15 min	^c^	^c^	16.5 ± 0.1
t 2 h	^c^	^c^	32.9 ± 2.0
t 6 h	^c^	^c^	31.7 ± 2.4
t 24 h	^b^	^b^	^b^
t 48 h	^b^	^b^	^b^
5-*O*-Caffeoylquinic acid			
t 15 min	^c^	^c^	11.1 ± 1.3
t 2 h	^c^	^c^	6.8 ± 0.5
t 6 h	^c^	^c^	^c^
t 24 h	^c^	^c^	^c^
t 48 h	^c^	^c^	^c^
Phenylpropanoic acids			
3-(3′-Hydroxyphenyl)propanoic acid			
t 15 min	^b^	^b^	^b^
t 2 h	29.7 ± 9.0	2.1 ± 1.1	30.9 ± 2.9
t 6 h	226.0 ± 12.9	230.9 ± 11.9	637.4 ± 39.3
t 24 h	309.6 ± 28.7	405.0 ± 75.3	1171.1 ± 41.8
t 48 h	285.8 ± 35.6	462.8 ± 14.6	1457.7 ± 49.3
3-(3′,4′-Dihydroxyphenyl)propanoic acid			
t 15 min	^b^	^b^	^b^
t 2 h	53.7 ± 6.2	49.0 ± 5.3	117.1 ± 5.8
t 6 h	197.5 ± 14.8	199.2 ± 17.5	315.8 ± 17.5
t 24 h	372.6 ± 62.0	466.9 ± 41.6	645.3 ± 95.1
t 48 h	485.1 ± 35.7	458.2 ± 70.8	529.6 ± 41.8
Phenylacetic acids			
Phenylacetic acid^a^			
t 15 min	105.9 ± 7.7	180.3 ± 6.0	440.8 ± 24.5
t 2 h	79.1 ± 11.2	150.0 ± 9.0	539.3 ± 49.6
t 6 h	^b^	^b^	^b^
t 24 h	^b^	^b^	^b^
t 48 h	^b^	^b^	^b^
Phenyl-γ-valerolactones			
5-(3′,4′-Dihydroxyphenyl)-γ-valerolactone			
t 15 min	^c^	^c^	^c^
t 2 h	^c^	^c^	^c^
t 6 h	^c^	^c^	^c^
t 24 h	15.5 ± 0.5	26.9 ± 0.3	136.7 ± 17.6
t 48 h	19.8 ± 0.5	38.2 ± 2.2	103.9 ± 4.3
5-(3′-Hydroxyphenyl)-γ-valerolactone			
t 15 min	^b^	^b^	^b^
t 2 h	^b^	^b^	^b^
t 6 h	^b^	^b^	^c^
t 24 h	1.4 ± 0.0	^c^	140.1 ± 11.2
t 48 h	2.0 ± 0.0	^c^	87.8 ± 5.9
5-Phenylvaleric acids			
5-(3′,4′-Dihydroxyphenyl)valeric acid			
t 15 min	^b^	7.1 ± 0.0	7.4 ± 0.0
t 2 h	^b^	7.6 ± 0.1	7.3 ± 0.3
t 6 h	7.6 ± 0.3	7.7 ± 0.4	7.6 ± 0.2
t 24 h	9.9 ± 2.5	8.5 ± 0.2	10.9 ± 0.8
t 48 h	10.0 ± 2.7	8.8 ± 0.3	13.1 ± 0.8
Phenylethanols			
2-(3′,4′-Dihydroxyphenyl)ethanol			
t 15 min	^b^	^b^	^b^
t 2 h	^b^	^b^	^b^
t 6 h	^c^	^c^	^c^
t 24 h	82.5 ± 0.5	80.1 ± 1.1	83.6 ± 1.5
t 48 h	98.9 ± 5.1	101.6 ± 11.8	108.0 ± 7.2
FLAVONOIDS			
Flavan-3-ols			
(−)-Epicatechin			
t 15 min	19.8 ± 2.8	18.0 ± 2.9	115.7 ± 19.0
t 2 h	14.4 ± 1.8	23.3 ± 2.9	195.4 ± 14.2
t 6 h	6.9 ± 0.5	10.9 ± 0.4	31.5 ± 0.9
t 24 h	^b^	8.1 ± 0.6	9.0 ± 1.4
t 48 h	^b^	^b^	8.6 ± 0.4
(+)-Catechin			
t 15 min	14.6 ± 2.0	12.8 ± 0.8	61.6 ± 10.6
t 2 h	16.3 ± 2.2	17.2 ± 1.7	109.8 ± 5.1
t 6 h	^c^	10.1 ± 0.6	27.0 ± 2.7
t 24 h	^c^	^c^	^c^
t 48 h	^c^	^c^	^c^
Procyanidin B_2_			
t 15 min	^b^	^c^	35.3 ± 4.9
t 2 h	^b^	^c^	36.3 ± 4.0
t 6 h	^b^	^b^	7.8 ± 0.4
t 24 h	^b^	^b^	^b^
t 48 h	^b^	^b^	^b^
Procyanidin B_1_			
t 15 min	3.5 ± 0.6	3.8 ± 1.1	12.7 ± 1.5
t 2 h	^b^	^c^	9.8 ± 0.6
t 6 h	^b^	^b^	^c^
t 24 h	^b^	^b^	^b^
t 48 h	^b^	^b^	^b^
Procyanidin C_1_			
t 15 min	^b^	^c^	4.1 ± 0.1
t 2 h	^b^	^c^	8.4 ± 1.2
t 6 h	^b^	^b^	4.2 ± 0.5
t 24 h	^b^	^b^	^b^
t 48 h	^b^	^b^	^b^
Flavonols			
Kaempferol			
t 15 min	37.5 ± 1.8	34.4 ± 1.6	47.8 ± 6.5
t 2 h	44.6 ± 5.1	51.1 ± 0.6	62.5 ± 4.4
t 6 h	13.5 ± 3.4	22.6 ± 2.1	26.9 ± 0.8
t 24 h	^c^	^c^	^c^
t 48 h	^c^	^c^	^c^
Quercetin-3-*O*-xyloside			
t 15 min	8.7 ± 0.3	5.5 ± 0.2	10.4 ± 1.2
t 2 h	^c^	^c^	^c^
t 6 h	^b^	^b^	^b^
t 24 h	^b^	^b^	^b^
t 48 h	^b^	^b^	^b^
Quercetin-3-*O*-rhamnoside			
t 15 min	57.1 ± 4.0	45.0 ± 1.9	61.1 ± 7.7
t 2 h	47.5 ± 1.4	42.7 ± 4.4	51.7 ± 1.8
t 6 h	12.5 ± 0.6	18.1 ± 1.3	16.5 ± 0.6
t 24 h	^c^	^c^	^c^
t 48 h	^b^	^c^	^c^
Flavones			
Apigenin-6,8-*C*-diglucoside			
t 15 min	3.4 ± 0.2	^c^	^c^
t 2 h	3.4 ± 0.2	^c^	^c^
t 6 h	^c^	^c^	^c^
t 24 h	^c^	^c^	^c^
t 48 h	^b^	^b^	^b^
Luteolin			
t 15 min	64.4 ± 2.5	52.2 ± 4.1	78.1 ± 2.4
t 2 h	63.8 ± 7.7	71.2 ± 8.3	93.5 ± 8.4
t 6 h	41.1 ± 5.9	52.7 ± 0.9	68.8 ± 2.7
t 24 h	^c^	^c^	^c^
t 48 h	^c^	^c^	^c^
Diosmetin			
t 15 min	10.8 ± 0.7	7.3 ± 0.2	9.5 ± 0.1
t 2 h	9.6 ± 1.2	8.4 ± 0.8	10.1 ± 1.0
t 6 h	^c^	6.9 ± 0.1	7.2 ± 0.1
t 24 h	^c^	^c^	^c^
t 48 h	^c^	^c^	^c^
Flavanones			
Naringenin			
t 15 min	905.6 ± 22.5	705.2 ± 8.9	980.0 ± 70.7
t 2 h	920.7 ± 100.6	1059.2 ± 148.8	1304.8 ± 146.6
t 6 h	508.8 ± 92.9	661.5 ± 7.9	871.0 ± 70.8
t 24 h	^c^	^c^	^c^
t 48 h	^c^	^c^	^c^
Eriodictyol			
t 15 min	80.8 ± 2.3	73.7 ± 0.0	155.3 ± 4.4
t 2 h	82.7 ± 11.3	97.0 ± 12.5	179.7 ± 17.3
t 6 h	45.7 ± 7.6	64.7 ± 0.5	109.3 ± 5.1
t 24 h	^c^	^c^	^c^
t 48 h	^c^	^c^	^c^
Eriodictyol-7-*O*-rutinoside			
t 15 min	25.8 ± 5.2	12.1 ± 0.4	56.7 ± 6.4
t 2 h	^c^	^c^	^c^
t 6 h	^c^	^b^	^c^
t 24 h	^b^	^b^	^b^
t 48 h	^b^	^b^	^b^
Hesperitin			
t 15 min	14.6 ± 1.0	10.3 ± 0.6	14.3 ± 0.8
t 2 h	10.4 ± 0.4	12.3 ± 1.7	15.2 ± 1.6
t 6 h	^c^	^c^	^c^
t 24 h	^c^	^c^	^c^
t 48 h	^c^	^c^	^c^
Hesperetin-7-*O*-rutinoside			
t 15 min	6.5 ± 1.5	3.6 ± 0.9	9.2 ± 0.3
t 2 h	^c^	^c^	^c^
t 6 h	^c^	^c^	^c^
t 24 h	^c^	^b^	^c^
t 48 h	^b^	^c^	^c^

a Data are expressed as mean ± standard deviation (nmol/g sample dm).

b Phenolic compounds derived from other sources and excluded for the total (poly)­phenols calculation.

c Not detected

d Below the limit of quantification

Although all volunteers were on a (poly)­phenol-free diet for the 48 h prior to the collection of the fecal sample, 6 (poly)­phenols (4-hydroxybenzoic acid, 2,5-dihydroxybenzoic acid, 3,4-dihydroxybenzoic acid, 3,4-dihydroxycinnamic acid, 3-(3′-hydroxyphenl)­propanoic acid, and phenylacetic acid) were still detected in the control sample containing fecal slurry without digested smoothies. These phenolics detected after colonic fermentation may not be exclusive to the degradation of (poly)­phenols present in the smoothie and could be derived from persistent catabolites not eliminated during the 48 h prior to fecal sample collection and from the microbial fermentation of aromatic amino acids after consumption of dietary proteins.^38,40^ Therefore, the content of these (poly)­phenolic compounds in fermented smoothies was corrected by subtracting the amounts found in the control samples.

The profiles of degradation of native (poly)­phenols and generation of microbial metabolites in untreated, HPP-treated, and HTST-treated smoothies during 48 h of *in vitro* fecal fermentation are shown in Figure 1A. Native (poly)­phenols were rapidly degraded before 24 h in all smoothie samples while a concomitant increase in catabolites was observed. From 24 h onward, only newly formed catabolites were detected, and their concentrations remained stable until the end of fermentation (48 h).

**Figure 1:**
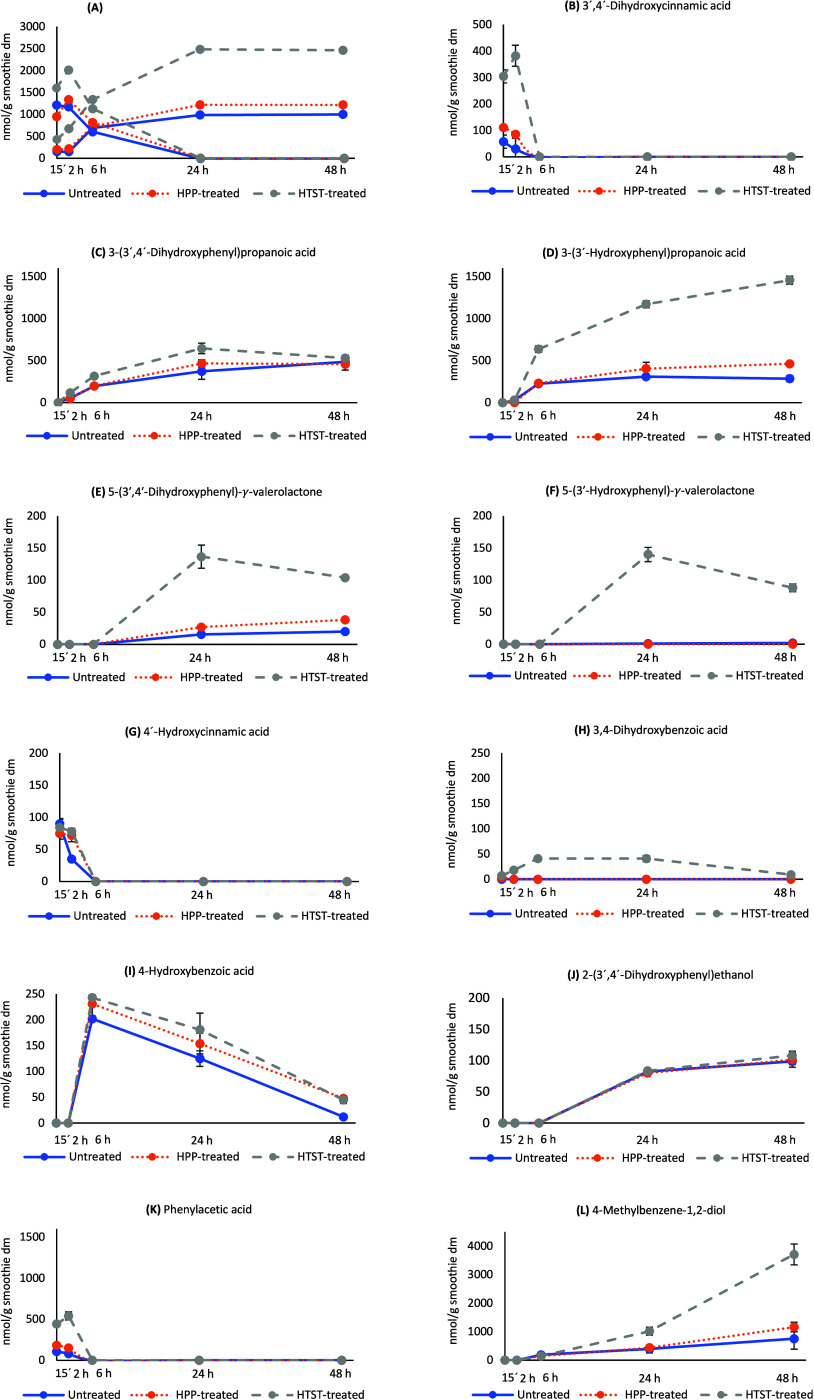
Kinetic profiles of degradation of native (poly)­phenols and generation of microbial metabolites (A), 3′,4′-dihydroxycinnamic acid (B), 3-(3′,4′-dihydroxyphenyl)­propanoic acid (C), 3-(3′-hydroxyphenyl)­propanoic acid (D), 5-(3′,4′-dihydroxyphenyl)-γ-valerolactone (E), 5-(3′-hydroxyphenyl)-γ-valerolactone (F), 4′-hydroxycinnamic acid (G), 3,4-dihydroxybenzoic acid (H), 4-hydroxybenzoic acid (I), 2-(3′,4′-dihydroxyphenyl)­ethanol (J), phenylacetic acid (K), and 4-methylbenzene-1,2-diol (L) in untreated (solid line), HPP-treated (dotted line), and HTST-treated (long dashed line) smoothies during *in vitro* fecal fermentation for 48 h.

The native (poly)­phenols quantified in the smoothies at the beginning of the fermentation (15 min) were hydroxycinnamic acids and flavonoids (flavan-3-ols, flavonols, flavones, and flavanones). Total hydroxycinnamic acids underwent rapid microbial degradation, already after 15 min, and were almost entirely degraded within 6 h of incubation (Table 3). Main representatives, 5-*O*-caffeoylquinic acid and 2-*O*-caffeoyl-3-(3′,4′-dihydroxyphenyl)­lactic acid, were found at very low concentration in HTST-treated sample and were even below the limit of quantification in HPP-treated and untreated smoothies at the very beginning of the fermentation (15 min) (Table 4), suggesting that microbial catabolism had already taken place during the first minutes of fermentation. Total flavonoids of the untreated smoothie remained stable after 2 h of fermentation, whereas HPP and HTST treatments increased flavonoids, reaching their maximum concentration at 2 h, rapidly declining after 6 h and being almost totally degraded at 24 h (Table 3). The increase observed after 2 h suggests that smoothies contained covalently bound flavonoids that were not determined in the initial samples (before and after GID) and were released at the beginning of colonic fermentation favored by the pasteurization process.^13^ Because of the intense microbial metabolism of native (poly)­phenols from smoothies, catabolites underwent a marked increase after 6 h of incubation due to the occurrence of a wide range of new phenolic compounds, especially in the HTST-treated smoothie (Figure 1A). New catabolites observed included the following non-flavonoids: benzene diols, benzoic acids, phenylpropanoic acids, phenylacetic acids, phenyl-γ-valerolactones, 5-phenylvaleric acids and phenylethanols (Table 3). Based on the concentration and time of appearance of the catabolites detected during fermentation, major and minor pathways for microbial metabolism of smoothie (poly)­phenols are proposed and illustrated in Figure 2.

**Figure 2:**
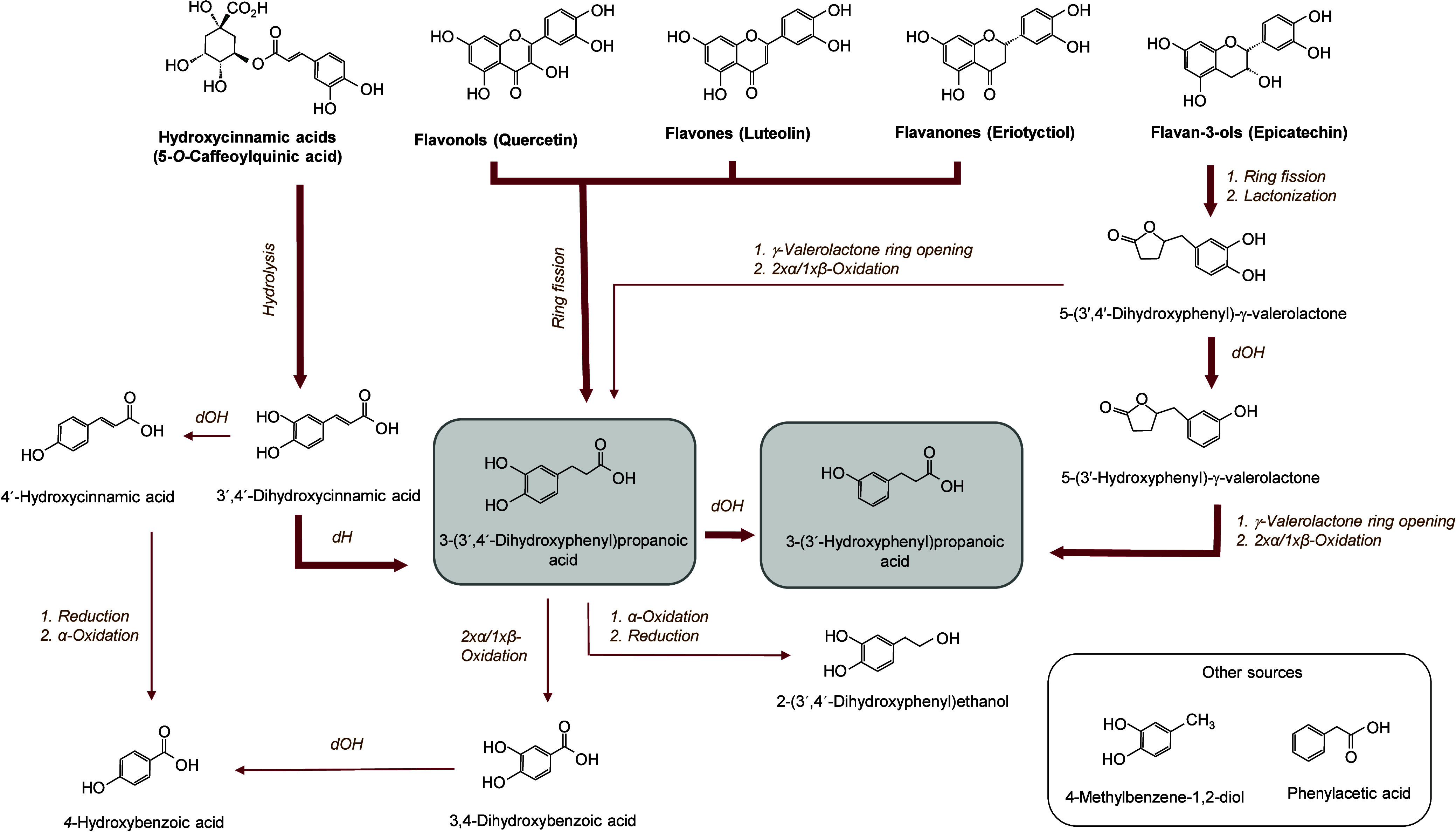
Major and minor proposed pathways for microbial metabolism of smoothie (poly)­phenols after *in vitro* colonic fermentation for 48 h. Bold arrows indicate the main catabolic routes. The two main end catabolites are marked with gray squares. Catabolites derived from other sources are marked with a white square.

The kinetics of intermediate and end catabolites from the main degradation pathways are illustrated in Figure 1B–F and comprised 3′,4′-dihydroxycinnamic acid, 3-(3′,4′-dihydroxyphenyl)­propanoic acid, 3-(3′-hydroxyphenyl)­propanoic acid, 5-(3′,4′-dihydroxyphenyl)-γ-valerolactone, and 5-(3′-hydroxyphenyl)-γ-valerolactone. 3′,4′-Dihydroxycinnamic acid was detected already at the beginning of the colonic fermentation as a transient peak disappearing after 6 h. This intermediate catabolite of hydroxycinnamic acids arises from the hydrolysis of 2-*O*-caffeoyl-3-(3′,4′-dihydroxyphenyl)­lactic acid^11^ and 5-*O*-caffeoylquinic acid^14^ catalyzed by microbial esterase, in line with the lower amounts of these native hydroxycinnamic acids found at the beginning of the fermentation. Next, 3-(3′,4′-dihydroxyphenyl)­propanoic acid was detected in increasing and high amounts for 24 h and remained constant until the end of the fermentation. This microbial catabolite may arise from 3′,4′-dihydroxycinnamic acid by the reduction of the aliphatic double bond^11^ as well as from C-ring fission of flavonols, flavones, and flavanones present in the smoothie.^41,42^ Subsequently, the 4-dehydroxylation of 3-(3′,4′-dihydroxyphenyl)­propanoic acid yielded 3-(3′-hydroxyphenyl)­propanoic acid, a known catabolic product of most (poly)­phenols^43^ which was together with its precursor one of the two main end catabolites. Among them, 3-(3′-hydroxyphenyl)­propanoic acid was found in much higher quantities in HTST-treated smoothie compared to untreated and HPP-treated smoothies, in line with the considerably higher amounts of native (poly)­phenols, especially 5-*O*-caffeoylquinic acid, after GID.

Flavan-3-ol concentration was highly reduced already at the beginning of the fermentation (15 min). Entering the colon, these flavonoids undergo C-ring opening leading to a diphenylpropan-2-ol intermediate.^44^ This intermediate catabolite was not quantified due to lack of standard, but mass spectrometer signals were detected at 2 h and peaked at 6 h (Figure S5). Diphenylpropan-2-ol intermediate is then transformed into 5-(3′,4′-dihydroxyphenyl)-γ-valerolactone which was detected below the limit of quantification at the beginning but increased notably at 24 h, especially in the HTST-treated smoothie due to the considerably higher amounts of flavan-3-ols present after GID. 5-(3′-Hydroxyphenyl)-γ-valerolactone was detected after 24 h, and similarly to its dihydroxy precursor, it increased significantly in the HTST-treated smoothie. Most probably phenyl-γ-valerolactones formed from 24 h onward were then further transformed to 3-(3′,4′-dihydroxyphenyl)­propanoic acid and ultimately to 3-(3′-hydroxyphenyl)­propanoic acid, which is consistent with the much higher amounts of these phenylpropanoics produced in HTST-treated smoothie. Concluding, the main catabolic routes of non-flavonoids as well as flavonoids present in the smoothies led to the formation of these two main end phenylpropanoic catabolites.

In addition, four other catabolites (Figure 1G–J) consistent with previous research on (poly)­phenol colonic catabolism^13,14,45–47^ were detected during colonic fermentation, which point to alternative minor pathways included in Figure 2. Aliphatic chain shortening of 3-(3′,4′-dihydroxyphenyl)­propanoic acid, via two consecutive α-oxidations or one β-oxidation, may lead to 3,4-dihydroxybenzoic acid which may further be dehydroxylated to 4-hydroxybenzoic. This latter may also have been formed from 3′,4′-dihydroxycinnamic acid by dehydroxylation leading to 4′-hydroxycinnamic acid, which then may suffer reduction of the double bond and chain shortening yielding 4-hydroxybenzoic acid.^37,48^ 2-(3′,4′-Dihydroxyphenyl)­ethanol was found after 24 h of incubation (Table 4) and might arise also from 3-(3′,4′-dihydroxyphenyl)­propanoic acid via α-oxidation of the acyl-chain leading to 3′,4′-dihydroxyphenylacetic acid (not detected intermediate) followed by reduction of the carboxylic group. The sum of all catabolites produced after 48 h of fermentation of smoothies (Table 3) was 999, 1216, and 2463 nmol/g dm for untreated, HPP-treated, and HTST-treated samples, respectively, and corresponds to 83–87% bioconversion of native (poly)­phenols into catabolites.

Figure 1K–L shows the kinetic profiles of two phenolic compounds, phenylacetic acid and 4-methylbenzene-1,2-diol, detected during fermentation but not included in the proposed pathway. Both showed clear kinetics consistent with its formation from the smoothie during colonic catabolism but may not arise from native (poly)­phenols present in the samples. Phenylacetic acid was already detected at the earliest stage of fermentation (15 min) in all smoothies (Table 4) but was completely degraded after 6 h. HTST processing promoted the formation of phenylacetic acid probably due to the cell wall softening effect of heat treatments enhancing the release of this compound.^13^ This low-molecular-weight metabolite was expected at later stages of fermentation, as Domínguez-Fernández et al.^14^ reported in Tudela artichokes, where phenylacetic acid peaked at 24 h and remained until 48 h of incubation as one of the major phenolic catabolites produced from hydroxycinnamic acids.

In the case of 4-methylbenzene-1,2-diol, this phenolic catabolite was detected after fermentation for 6 h and was the most abundant end product after 48 h. HTST processing seems to promote its formation to a greater extent compared to untreated and HPP-treated smoothies (Figure 1L). Although some studies^49^ detected 4-methylbenzene-1,2-diol during *in vitro* colonic fermentation of hydroxycinnamic acid and flavan-3-ol rich foods, it has been described as a minor catabolite of colonic degradation. The high quantities found in this study (Table 4 and Figure 1L), which surpass the total (poly)­phenol contents of the smoothies by far, suggest a different origin. Possible origins for these two potential microbial catabolites not included in the pathway might be the breakdown of aromatic amino acids from smoothie protein content.^40,50^ However, further research is needed to clarify their origins in the smoothies.

The changes in (poly)­phenolic profiles after *in vitro* GID, as well as along the different points of *in vitro* colonic fermentation, is depicted in Figure 3. The main representatives in the undigested smoothies, hydroxycinnamic acids and flavan-3-ols, were notably decreased after GID in all samples. Afterward, the changes in phenolic families during the first steps of colonic fermentation (from 15 min to 2 h) were less pronounced. After 6 h, native flavonoids were degraded by half, and the remaining (poly)­phenols were new catabolites generated. From 24 h onward, low-molecular-weight catabolites accounted for 100% of the total (poly)­phenol derivatives, principally phenylpropanoic acid and to a lesser extent benzoic acids. The (poly)­phenolic profile remained almost unchanged until the end of colonic fermentation (48 h). Regarding pasteurization processes, HTST treatment favored the generation of higher concentrations of gut-related metabolites due to the more than 2-fold higher bioaccessibility of (poly)­phenols compared to the untreated smoothie, especially in hydroxycinnamic acids and flavan-3-ols, resulting in higher amounts of 3-(3′-hydroxyphenyl)­propanoic acid and phenyl-γ-valerolactones.

**Figure 3:**
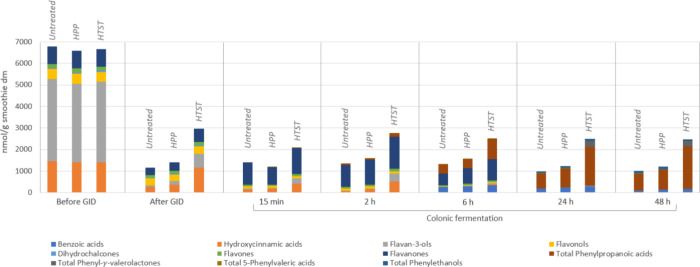
Changes in (poly)­phenolic profiles after *in vitro* GID and during *in vitro* fecal fermentation for 48 h in untreated, HPP-treated, and HTST-treated smoothies.

According to the changes observed in (poly)­phenolic profiles along the gastrointestinal tract, native (poly)­phenols present in the smoothie may be the main compounds responsible for potential health benefits in the upper gastrointestinal tract and in the colon during the initial stages before their breakdown to lower molecular weight compounds. These latter catabolites might exert health benefits at later stages of colonic transit or be absorbed and enter circulation to act on a systemic level. Research on potential health benefits of (poly)­phenols, specifically on the underlying mechanism of action, should take into account the conversions taking place in the gastrointestinal tract and consider native (poly)­phenols but most importantly intermediate and end catabolites. In this sense, results obtained so far on bioactivity of phenyl-γ-valerolactones, the intermediate flavan-3-ol catabolites, have shown promising results on inflammation markers, as recently reviewed by Mena et al.^48^ Similarly, the main end catabolites found in the present study, 3-(3′,4′-dihydroxyphenyl)­propanoic acid and 3-(3′-hydroxyphenyl)­propanoic acid, have demonstrated biological properties as antidiabetic, anti-inflammatory and chemopreventive agents.^51^

In conclusion, high-pressure (HPP) and thermal (HTST) pasteurization applied to smoothies improved (poly)­phenol bioaccessibility after gastrointestinal digestion and led to higher amounts of catabolites during colonic fermentation compared to the unprocessed smoothie, especially in the HTST-treated smoothie. Therefore, both pasteurization processes could be proposed as suitable preservation treatments for fruit and vegetable based smoothies in the food industry, in order to develop beverages that preserve native (poly)­phenols and gut-related metabolites and their further potential health benefits.

## Supplementary Material



## Notes

Figure S1: Chromatograms of untreated smoothie before and after *in vitro* digestion. Figure S2: Chromatograms of HPP-treated smoothie before and after *in vitro* digestion. Figure S3: Chromatograms of HTST-treated smoothie before and after *in vitro* digestion. Figure S4: Chromatograms for HTST-treated smoothie during *in vitro* fecal fermentation for 15 min, 2 h, 6 h, 24 h, and 48 h. Figure S5: Chromatogram for the diphenylpropan-2-ol intermediate (C_15_H_16_O_6_) in HTST-treated smoothie *in vitro* fecal fermentation for 15 min, 2 h, 6 h, 24 and 48 h. Table S1: Electrolyte stock solutions and simulated salivary, gastric, and intestinal fluids (SSF, SGF, and SIF) for *in vitro* gastrointestinal digestion. Table S2: Mass spectrometric parameters of (poly)­phenolic compounds analyzed by LC-MS/MS for bioaccessibility assessment. Table S3: Mass spectrometric parameters of (poly)­phenolic compounds analyzed by LC-HRMS for *in vitro* colonic fermentation. (Poly)­phenols are reported according to the nomenclature proposed by Kay et al.^18^ and other common names. (PDF)
