# Supplementary material for: High-Pressure and Thermal Pasteurization Applied to Smoothies Enhances (Poly)Phenol Bioaccessibility along the Gastrointestinal Tract
Source: J Agric Food Chem. 2025 Jun 11;73(25):15561–78. doi: 10.1021/acs.jafc.4c09166 (PMC12203591; doi:10.1021/acs.jafc.4c09166)
Supplement: Supplementary file 1 [file jf4c09166_si_001.pdf]

TITLE: High-pressure and thermal pasteurization applied to smoothies enhance (poly)phenols´ bioaccessibility along the gastrointestinal tract

AUTHORS: Cristina Matías[ab], Gema Pereira-Caro[cd], María-José Sáiz-Abajo[b], Concepción Cid[ae], Iziar A. Ludwig[ae*], and María-Paz De Peña[ae].

[a] Centre for Nutrition Research and Department of Nutrition, Food Science and Physiology, Faculty of Pharmacy and Nutrition, University of Navarra, Pamplona, 31008, Spain.

[b] National Centre for Food Technology and Safety (CNTA), San Adrián, 31570, Spain.

[c] Department of Agroindustry and Food Quality, Andalusian Institute of Agricultural and Fisheries Research and Training (IFAPA), Alameda del Obispo, Avda. Menéndez-Pidal, Córdoba, 14004, Spain.

[d] Foods for Health Group, Instituto Maimónides de Investigación Biomédica de Córdoba (IMIBIC), Córdoba, 14004, Spain.

[e] IdiSNA, Navarra Institute for Health Research, Pamplona, 31008, Spain.

* Corresponding Author: Iziar A. Ludwig: iludwig@unav.es; +34 948 425 600 (Ext. 806652)

SUPPLEMENTARY MATERIAL

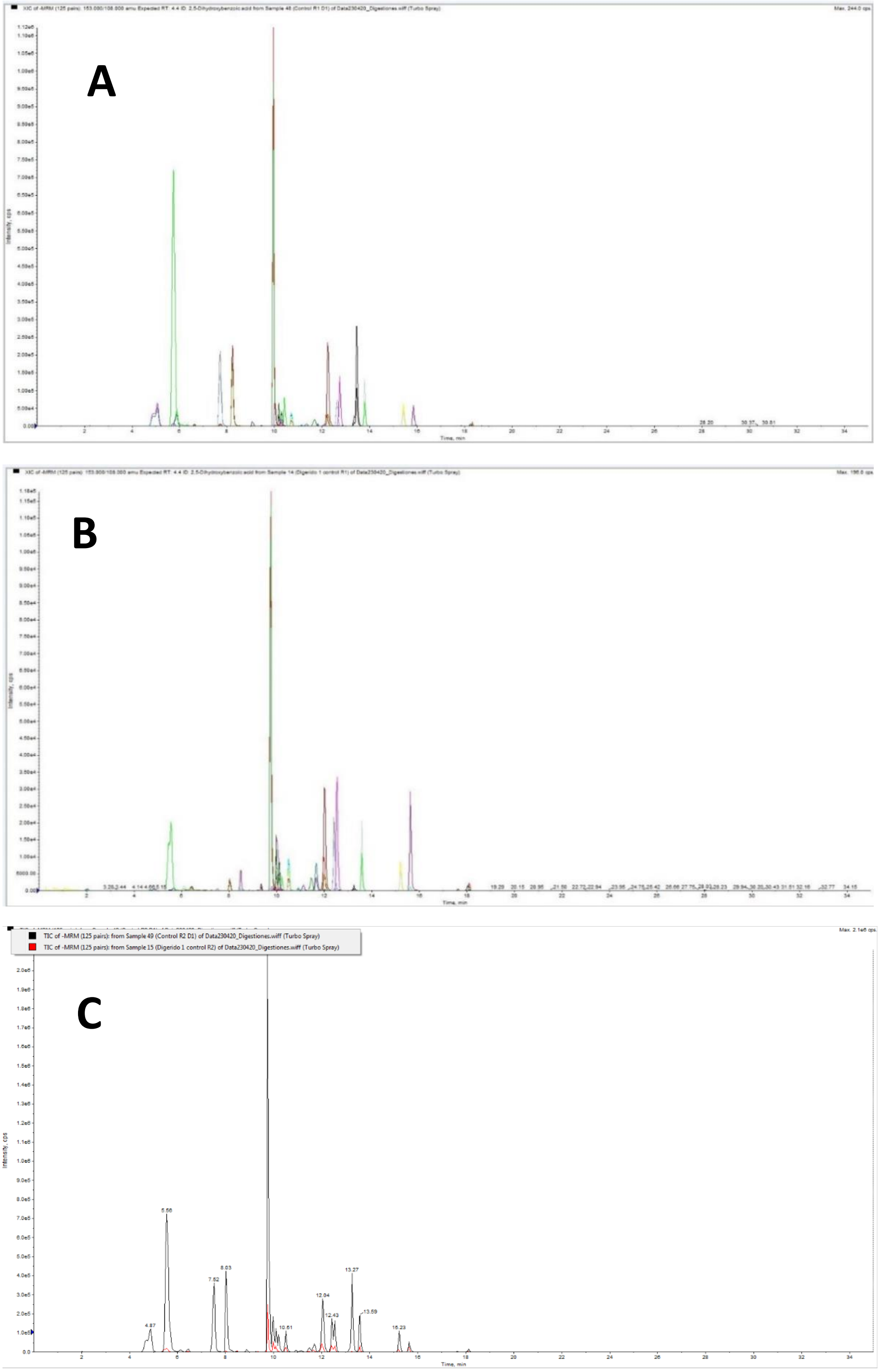

Figure S1. Chromatograms of untreated smoothie before and after *in vitro* digestion. A) Multiple extracted ion chromatogram (XIC) of undigested untreated smoothie. B) Multiple extracted ion chromatogram (XIC) of digested untreated smoothie. C) Overlayed total ion chromatograms of untreated smoothie before (black) and after digestion (red).

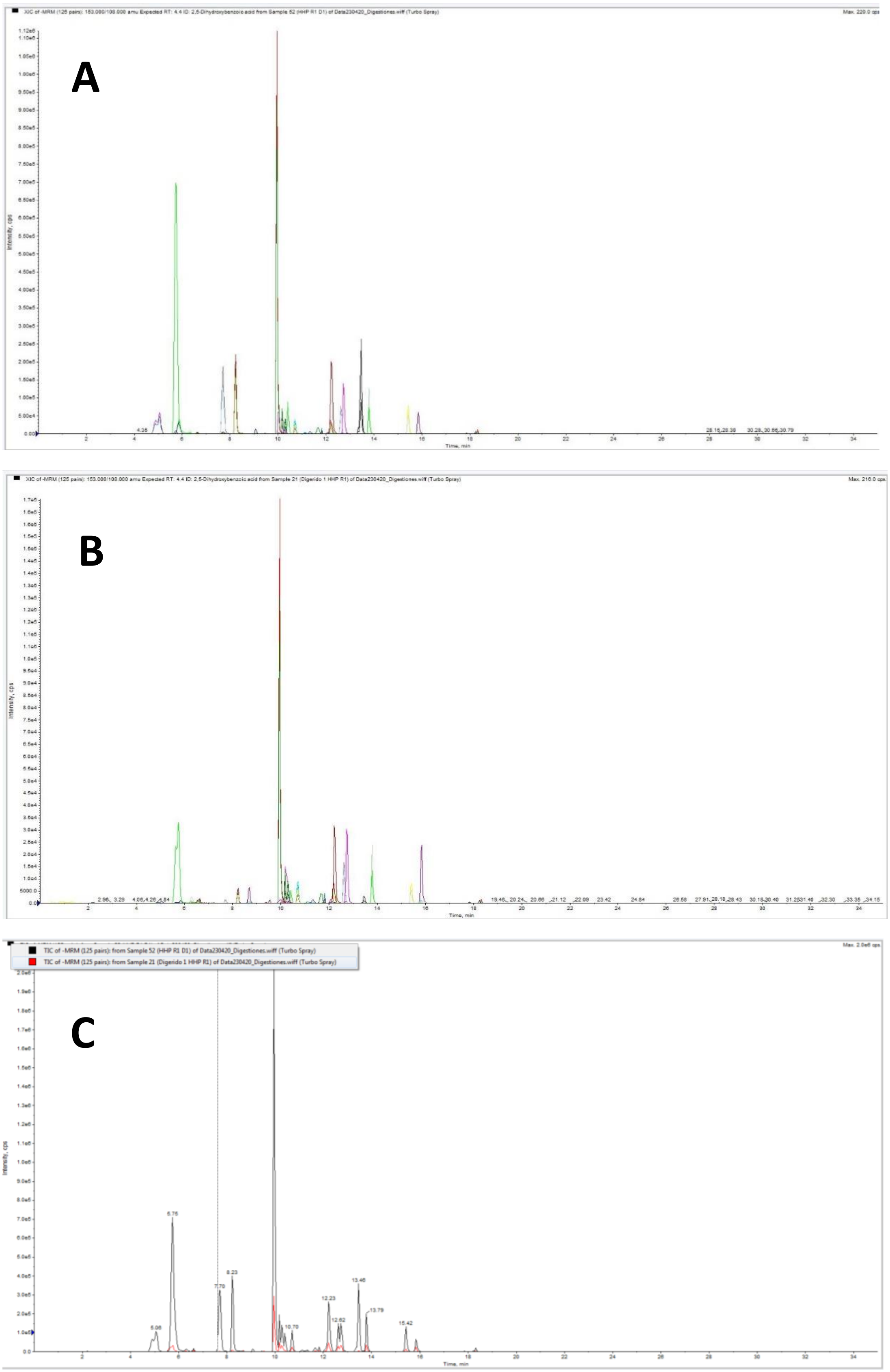

Figure S2. Chromatograms of HPP-treated smoothie before and after *in vitro* digestion. A) Multiple extracted ion chromatogram (XIC) of undigested HPP-treated smoothie. B) Multiple extracted ion chromatogram (XIC) of digested HPP-treated smoothie. C) Overlayed total ion chromatograms of HPP-treated smoothie before (black) and after digestion (red).

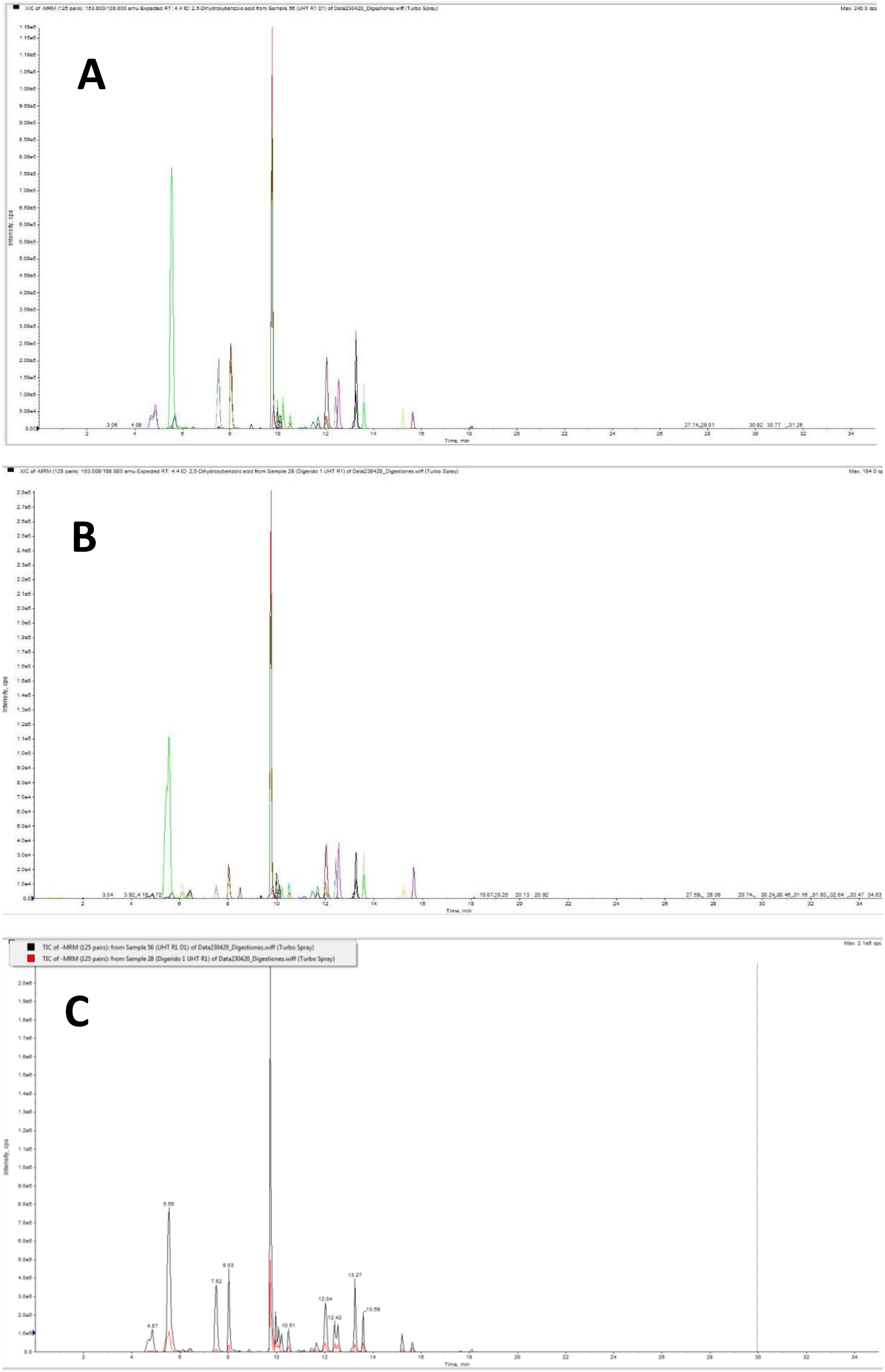

Figure S3. Chromatograms of HTST-treated smoothie before and after *in vitro* digestion. A) Multiple extracted ion chromatogram (XIC) of undigested HTST-treated smoothie. B) Multiple extracted ion chromatogram (XIC) of digested HTST-treated smoothie. C) Overlayed total ion chromatograms of HTST-treated smoothie before (black) and after digestion (red).

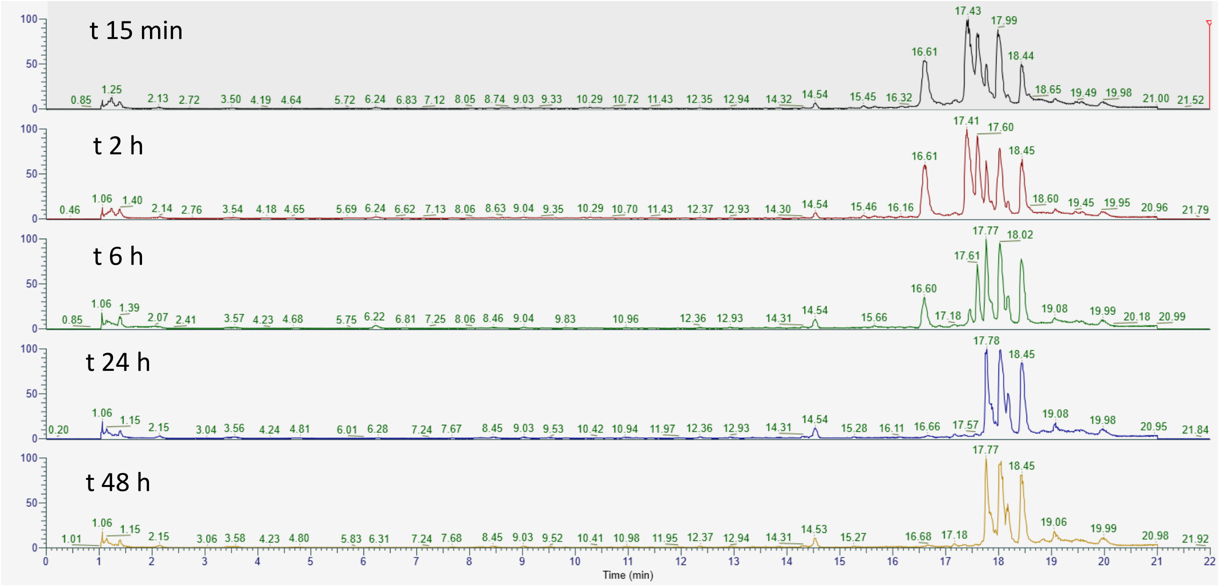

Figure S4. Chromatograms for HTST-treated smoothie during 15 min, 2 h, 6 h, 24 h and 48 h of *in vitro* faecal fermentation.

$C_{15}H_{16}O_6$

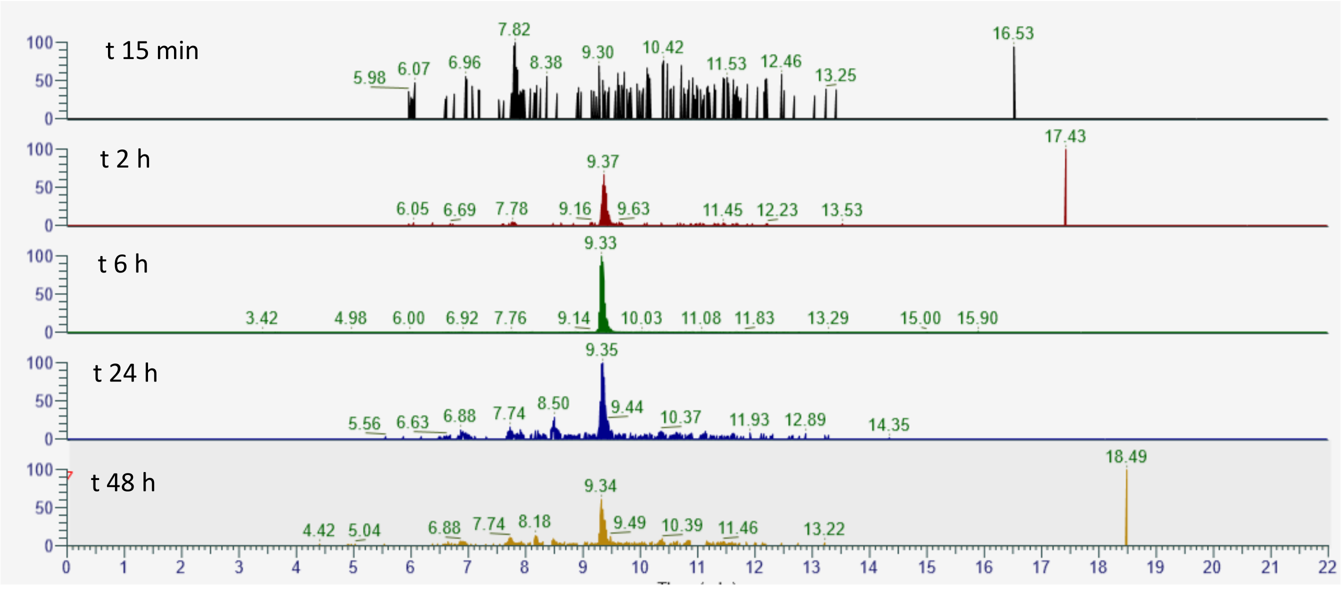

Figure S5. Chromatogram for the diphenylpropan-2-ol intermediate ($C_{15}H_{16}O_6$) in HTST-treated smoothie during 15 min, 2 h, 6 h, 24 h and 48 h of *in vitro* faecal fermentation.

Table S1. Electrolyte stock solutions and simulated salivary, gastric and intestinal fluids (SSF, SGF and SIF) for *in vitro* gastrointestinal digestion.

	Stock concentrations		SSF	SGF	SIF
Salt solution	(g/L)	(mol/L)	Volume (mL) of stock solutions for the preparation of 500 mL		
KCl	37.28	0.50	18.88	8.63	8.50
KH_2PO_4	68.05	0.50	4.63	1.13	1.00
$NaHCO_3$	84.01	1.00	8.50	15.63	53.13
NaCl	116.80	2.00	-	14.75	12.00
$MgCl_2(H_2O)_6$	30.48	0.15	0.63	0.50	1.38
$(NH_4)_2CO_3$	48.05	0.50	0.08	0.63	-
HCl	-	6.00	0.11	1.63	0.88
$CaCl_2(H_2O)_2$	44.11	0.30	3.13	0.31	1.25

Table S2. Mass spectrometric parameters of (poly)phenolic compounds analysed by LC-MS/MS for bioaccessibility assessment. (Poly)phenols are reported according to the nomenclature proposed by Kay et al. (18) and other common names.

Recommended name	Other common names	Rt (min)	$[M-H]^-$ (m/z)	MS/MS fragmentation	CE (eV)
**NON-FLAVONOIDS**					
**Benzoic acids**					
2,5-Dihydroxybenzoic acid	-	4.4	153	108, 109	-30
3,4-Dihydroxybenzoic acid	Protocatechuic acid	2.2	153	109, 91, 81	-35
3,4,5-Trihydroxybenzoic acid	Gallic acid	0.9	169	125	-20
3,5-Dimethoxy-4-hydroxybenzoic acid	Syringic acid	6.5	197	153, 182	-16
**Hydroxycinnamic acids**					
4´-Hydroxycinnamic acid	*p*-coumaric	8.7	163	119, 93	-30
3´,4´-Dihydroxycinnamic acid	Caffeic acid	6.1	179	135, 134	-30
4´-Hydroxy-3´-methoxycinnamic acid	Ferulic acid	9.5	193	134, 178	-20
2-*O*-Caffeoyl-3-(3´,4´-dihydroxyphenyl)lactic acid	Rosmarinic acid	13.3	359	161, 197	-22
2,3-*O*-Dicaffeoyltartaric acid	Chicoric acid	9.9	473	149, 135	-26
5-*O*-Caffeoylquinic acid	Chlorogenic acid	5.5	353	191, 179	-20
4-*O*-Caffeoylquinic acid	Cryptochlorogenic acid	6.4	353	173, 179	-30
3,5-*O*-Dicaffeoylquinic acid	-	12.1	515	353, 191	-30
3,4-*O*-Dicaffeoylquinic acid	-	11.6	515	353, 335, 299	-30
4,5-*O*-Dicaffeoylquinic acid	-	13.2	515	353, 173	-30
**Phenylethanols**					
2-(3´,4´-Dihydroxyphenyl)ethanol	Hydroxytyrosol	2.1	153	123, 108	-30
**FLAVONOIDS**					
**Flavan-3-ols**					
(-)-Epicatechin	-	5.7	289	245, 109	-18
(+)-Catechin	-	8.2	289	245, 109	-18
Epigallocatechin	-	5.1	30	125, 179	-28
Procyanidin B_2 ((-)-epicatechin, (-)-epicatechin)	-	7.5	577	289, 125	-32
Procyanidin B_1 ((-)-epicatechin, (+)-catechin)	-	4.9	577	289, 125	-32
Procyanidin C_1 ((-)-epicatechin, (-)-epicatechin, (-)-epicatechin)	-	8.9	865	125, 287	-76
**Flavonols**					
Kaempferol	-	18.1	285	93, 65	-50
Kaempferol-7-*O*-glucoside	-	12.3	447	285, 284, 151	-30
Kaempferol-3-*O*-glucuronide	-	12.0	461	285, 113	-28
Kaempferol-3-*O*-rutinoside	-	11.0	593	285, 255, 227	-50
Quercetin	-	15.7	301	151, 179	-30
Quercetin-3-*O*-arabinoside	Guaiaverin	10.9	433	300, 271	-32
Quercetin-3-*O*-xyloside	Reynoutrin	10.5	433	300, 301	-30
Quercetin-3-*O*-rhamnoside	Quercitrin	12.0	447	301, 283	-40
Quercetin-3-*O*-glucoside	Isoquercitrin	10.1	463	300, 301, 271	-35
Quercetin-3-*O*-galactoside	Hyperoside	9.9	463	300, 301	-36

Table S2. (Continued)

Recommended name	Other common names	Rt (min)	[M-H]- (m/z)	MS/MS fragmentation	CE (eV)
Quercetin-3-*O*-rutinoside	Rutin	9.8	609	301, 179	-50
Quercetin-3-*O*-glucoside	Isoquercitrin	10.1	463	300, 301, 271	-35
Quercetin-3-*O*-galactoside	Hyperoside	9.9	463	300, 301	-36
Quercetin-3-*O*-rutinoside	Rutin	9.8	609	301, 179	-50
Isorhamnetin	-	18.2	315	300, 151	-30
Isorhamnetin-3-*O*-glucoside	-	12.2	477	314, 271, 285	-25
Isorhamnetin-3-*O*-rutinoside	-	11.5	623	577, 315	-30
**Dihydrochalcones**					
Phloretin	-	17.9	273	167, 123	-22
Phloretin-2´-*O*-glucoside	Phloridzin	13.6	435	273, 167	-22
**Flavones**					
Apigenin	-	17.6	269	151, 149, 117	-30
Apigenin-7-*O*-glucoside	-	12.3	431	269, 311, 283	-40
Apigenin-7-*O*-glucuronide	-	12.7	445	269, 113, 85	-30
Apigenin-7-*O*-rutinoside	Isorhoifolin	11.5	577	269	-40
Apigenin-7-(2-*O*-apiosylglucoside)	Apiin	12.0	563	269, 118	-50
Apigenin-8-*C*-glucoside	Vitexin	9.8	431	311, 283	-30
Apigenin-6,8-*C*-diglucoside	Vicenin-2	8.4	593	353, 383, 473	-45
Luteolin	-	15.6	285	133, 151	-50
Luteolin-7-*O*-glucoside	Cynaroside	10.2	447	285, 327	-30
Luteolin-8-*C*-glucoside	Orientin	9.4	447	327, 297, 285	-35
Luteolin-7-*O*-glucuronide	-	10.2	461	285, 327	-30
Diosmetin	-	18.1	299	284, 256	-40
Diosmetin-7-*O*-glucoside	-	13.1	461	299, 284	-35
Diosmetin-7-*O*-rutinoside	Diosmin	12.4	607	299, 284	-34
**Flavanones**					
Naringenin-7-*O*-rutinoside	Narirutin	11.1	579	271, 151	-30
Isosakuranetin-7-*O*-rutinoside	Didymin	15.6	593	285, 309	-30
Eriodictyol	-	15.3	287	151, 135	-20
Eriodictyol-7-*O*-rutinoside	Eriocitrin	9.8	595	287, 151	-32
Hesperetin	-	18.1	301	164, 108	-32
Hesperetin-7-*O*-rutinoside	Hesperedin	12.5	609	301, 342	-30

Rt, retention time; m/z, mass-to-charge ratio; [M–H]⁻, Negatively charged molecular ion; CE, Collision energy.

Table S3. Mass spectrometric parameters of (poly)phenolic compounds analysed by LC-HRMS for *in vitro* colonic fermentation. (Poly)phenols are reported according to the nomenclature proposed by Kay et al. (18) and other common names.

Recommended name	Other common names	Rt (min)	$[M-H]^-$ (m/z)	Δ m/z (ppm)	LoQ (ng/mL)
**NON-FLAVONOIDS**					
**Benzene diols and triols**					
4-Methylbenzene-1,2-diol	4-Methycatechol	5.8	123.04515	0.68820	
Benzene-1,3,5-triol	Phloroglucinol	2.2	125.02442	0.37967	
Benzene-1,2,3-triol	Pyrogallol	2.5	125.02442	0.07456	
**Benzaldehydes**					
4-Hydroxy-3-methoxybenzaldehyde	Vanillin	9.5	151.04007	-0.20483	
**Benzoic acids**					
4-Hydroxybenzoic acid	-	6.9	137.02442	0.29074	
3-Hydroxybenzoic acid	-	8.3	137.02442	-0.15469	
2,5-Dihydroxybenzoic acid	-	6.9	153.01933	0.81660	
3,4-Dihydroxybenzoic acid	Protocatechuic acid	5.0	153.01933	0.71689	
3,4,5-Trihydroxybenzoic acid	Gallic acid	2.8	169.01425	0.28095	25
3,5-Dimethoxy-4-hydroxybenzoic acid	Syringic acid	8.9	197.04555	0.29699	
**Hydroxycinnamic acids**					
4´-Hydroxycinnamic acid	*p*-coumaric	10.3	163.04007	-0.00258	25
3´,4´-Dihydroxycinnamic acid	Caffeic acid	8.7	179.03498	0.64353	
4´-Hydroxy-3´-methoxycinnamic acid	Ferulic acid	10.9	193.05063	0.15115	
4’-Hydroxy-3’,5’-dimethoxycinnamic	Sinapic acid	11.0	223.06120	0.62912	
2-O-Caffeoyl-3-(3´,4´-dihydroxyphenyl)lactic acid	Rosmarinic acid	12.6	359.07724	0.67988	10
5-*O*-Caffeoylquinic acid	Chlorogenic acid	8.5	353.08781	1.00622	10
4-*O*-Caffeoylquinic acid	Cryptochlorogenic acid	8.5	353.08781	0.48763	
3,5-*O*-Dicaffeoylquinic acid	-	11.9	515.11950	0.84268	
3,4-*O*-Dicaffeoylquinic acid	-	12.0	515.11950	0.36873	
4,5-*O*-Dicaffeoylquinic acid	-	12.6	515.11950	0.25025	
**Phenylpropanoic acids**					
3-(3´-Hydroxyphenyl)propanoic acid	-	10.4	165.05572	0.12333	
3-(3´,4´-Dihydroxyphenyl)propanoic acid	Dihydrocaffeic acid	7.8	181.05063	0.16117	
3-(4´-Hydroxy-3´-methoxyphenyl)propanoic acid	Dihydroferulic acid	10.2	195.06628	0.41255	
3-(3´-Hydroxy-4´-methoxyphenyl)propanoic acid	Dihydroisoferulic acid	11.2	195.06628	0.49077	
**Phenylacetic acids**					
Phenylacetic acid	-	8.7	135.04515	0.57056	
3´,4´-Dihydroxyphenylacetic acid	DOPAC	15.4	167.03498	-0.40645	
4´-Hydroxy-3´-methoxyphenylacetic acid	Homovanillic acid	9.4	181.05063	0.66684	
**Phenyl-*γ*-valerolactones**					
5-(3’,4’-Dihydroxyphenyl)-*γ*-valerolactone	-	9.3	207.06628	0.53602	10
5-(3’-Hydroxyphenyl)-*γ*-valerolactone	-	11.5	191.07137	0.05556	50
5-(4’-Hydroxyphenyl)-*γ*-valerolactone	-	11.8	191.07137	0.21527	

Table S3. (Continued)

Recommended name	Other common names	Rt (min)	$[M-H]^-$ (m/z)	Δ m/z (ppm)	LoQ (ng/mL)
**5-Phenylvaleric acids**					
5-(3′-Hydroxyphenyl)valeric acid	-	13.8	193.08702	-0.31158	
5-(3′,4′-Dihydroxyphenyl)valeric acid	-	11.8	209.08193	0.41128	
**Phenylethanols**					
2-(3´,4´-Dihydroxyphenyl)ethanol	Hydroxytyrosol	5.0	153.05572	0.13300	50
**FLAVONOIDS**					
**Flavan-3-ols**					
(-)-Epicatechin	-	9.3	289.07176	0.37671	
(+)-Catechin	-	8.0	289.07176	1.22128	10
Epigallocatechin	-	7.6	305.06668	0.70323	
Procyanidin B_2	-	8.9	577.13515	1.44910	10
Procyanidin B_1	-	7.5	577.13515	-0.24298	10
Procyanidin C_1	-	9.8	865.19854	1.41940	10
**Flavonols**					
Kaempferol	-	15.6	285.04046	0.34332	10
Kaempferol-3-*O*-glucuronide	-	12.3	461.07255	1.23650	
Kaempferol-3-*O*-rutinoside	-	11.9	593.15119	1.01902	
Quercetin	-	14.2	301.03538	-0.23253	
Quercetin-3-*O*-arabinoside	Guaiaverin	12.0	433.07763	0.64971	
Quercetin-3-*O*-xyloside	Reynoutrin	11.8	433.07763	0.43831	10
Quercetin-3-*O*-rhamnoside	Quercitrin	12.3	447.09328	0.35787	10
Quercetin-3-*O*-glucoside	Isoquercitrin	11.5	463.08820	1.30893	
Quercetin-3-*O*-galactoside	Hyperoside	11.3	463.08820	1.77024	
Quercetin-3-*O*-rutinoside	Rutin	11.2	609.14611	1.21578	
Isorhamnetin	-	15.8	315.05103	0.66343	
Isorhamnetin-3-*O*-glucoside	-	12.3	477.10385	0.77033	
Isorhamnetin-3-*O*-rutinoside	-	12.1	623.16176	0.95243	
**Dihydrochalcones**					
Phloretin	-	15.4	273.07685	0.31059	
Phloretin-2´-*O*-glucoside	Phloridzin	12.7	435.12967	0.90989	
**Flavones**					
Apigenin	-	15.5	269.04555	0.16080	
Apigenin-7-*O*-glucoside	-	12.4	431.09837	0.60963	
Apigenin-7-*O*-glucuronide	-	12.6	445.07763	0.22080	
Apigenin-7-*O*-rutinoside	Isorhoifolin	12.1	577.15628	0.79403	
Apigenin-7-(2-*O*-apiosylglucoside)	Apiin	12.2	563.14063	0.74981	
Apigenin-8-*C*-glucoside	Vitexin	11.0	431.09837	0.18489	
Apigenin-6,8-*C*-diglucoside	Vicenin-2	9.7	593.15119	1.32772	10
Luteolin	-	14.3	285.04046	0.23626	25
Luteolin-7-*O*-glucoside	Cynaroside	11.5	447.09328	0.84646	
Luteolin-8-*C*-glucoside	Orientin	10.5	447.09328	1.05123	

Table S3. (Continued)

Recommended name	Other common names	Rt (min)	$[M\text{-}H]^-$ (m/z)	Δ m/z (ppm)	LoQ (ng/mL)
Luteolin-7-*O*-glucuronide	-	11.7	461.07255	2.36170	
Diosmetin	-	15.8	299.05611	0.24364	10
Diosmetin-7-*O*-glucoside	-	12.8	461.10893	1.09743	
Diosmetin-7-*O*-rutinoside	Diosmin	12.4	607.16684	1.55747	
**Flavanones**					
Naringenin	-	15.2	271.06076	0.85547	50
Naringenin-7-*O*-rutinoside	Narirutin	11.8	579.17193	0.74810	
Isosakuranetin-7-*O*-rutinoside	Didymin	14.2	593.18758	0.27671	
Eriodictyol	-	13.8	287.05611	0.25382	25
Eriodictyol-7-*O*-rutinoside	Eriocitrin	11.0	595.16684	0.97356	10
Hesperitin	-	15.6	301.07176	0.46306	10
Hesperetin-7-*O*-rutinoside	Hesperedin	12.3	609.18249	1.51128	10

Rt, retention time; m/z, mass-to-charge ratio; $[M\text{–}H]^-$, Negatively charged molecular ion; Δ m/z (ppm), difference between experimental mass and theoretical mass; LoQ (ng/mL), limit of quantification.
